# Tuning and tracking the growth of gold nanoparticles synthesized using binary surfactant mixtures†

**DOI:** 10.1039/d0na00214c

**Published:** 2020-03-25

**Authors:** Karthik Raghunathan, Jibin Antony, Sarmad Munir, Jens-Petter Andreassen, Sulalit Bandyopadhyay

**Affiliations:** Ugelstad Laboratory, Department of Chemical Engineering, Norwegian University of Science and Technology N-7491 Trondheim Norway; Department of Chemical Engineering, Norwegian University of Science and Technology N-7491 Trondheim Norway sulalit.bandyopadhyay@ntnu.no; Yara International ASA, Herøya Research Park Hydrovegen 67 3936 Porsgrunn Norway

## Abstract

Synthesis of gold nanorods (Au NRs) using surfactant-mediated seeded growth involves the interplay of parameters such as pH, reducing agent, and surfactant among others. The use of binary surfactant mixtures of cetyltrimethylammonium bromide (CTAB) and oleic acid (OA) has been reported by our group previously to obtain other anisotropic shapes. However, there are no reports investigating the growth kinetics and mechanisms of such shapes. Here, we report for the first time a ternary representation for compact visualization of shape transitions of gold nanoparticles (Au NPs) as a function of reaction parameters. Further, using UV-Vis spectrophotometry, the growth kinetics of these shapes was tracked using an in-house developed technique. The interplay between the experimental parameters and the properties of Au NPs was investigated using statistical analysis which showed that the reducing agent and pH were significant in influencing shape and growth kinetics. We further propose a growth mechanism in which the supersaturation of growth units controls the final shapes obtained.

## Abstract 2

The effects of synthesis parameters on the shape of gold nanoparticles (Au NPs) and their growth are investigated. A ternary space to study their shape transitions is reported for the first time. A growth mechanism based on supersaturation is proposed.

## Graphical abstract

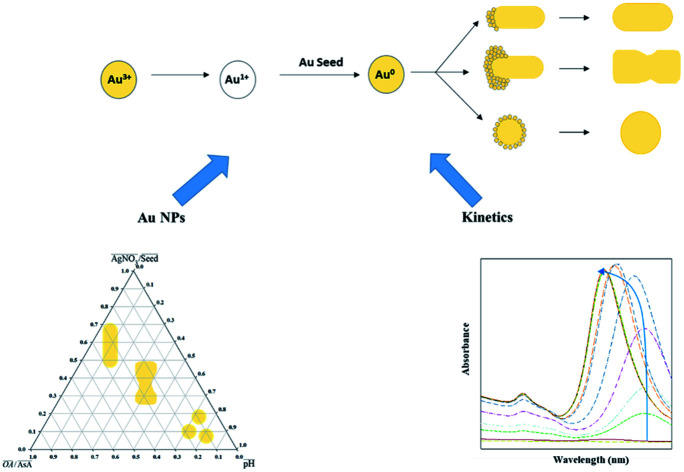

## Introduction

Gold nanoparticles (Au NPs) have been a topic of intensive research owing to their remarkable plasmonic properties, biocompatibility, enhanced stability and low cytotoxicity.^1–5^ The plasmonic properties of Au NPs depend on their size, shape, dielectric properties and the local environment around the NPs,^6^ and hence fine-tuning one or a combination of these leads to applications that can range from bio-sensing, photothermal therapy, targeted drug delivery and catalysis among others.^1,7–12^ With an aim to achieve control of these properties and in turn of the final applications, it is essential to understand and control the growth of Au NPs.

Among other synthesis methods, seed-mediated growth in the presence of cetyltrimethylammonium bromide (CTAB), developed by Jana *et al.*,^13^ has garnered considerable interest among researchers since its inception, due to the ease and robustness with which it can induce shape control. This method involves the growth of NPs in supersaturated growth solutions by introducing a seed solution. A number of modifications have been employed in this method in order to achieve better shape selectivity. Nikoobakht *et al.* performed experiments by adjusting the silver content of the growth solution in order to obtain nanorods (NRs) with controlled Aspect Ratios (ARs).^14^ The pH of the growth solution, being an important parameter in determining the reduction potential of the reducing agent (ascorbic acid (AsA)), has been shown to have a significant effect on the shape of Au NPs.^15,16^ Studies performed by Okitsu *et al.* report a substantial decrease in the AR of Au NRs with an increase in the pH of the growth solution, with shapes resembling spheres at pH 9.8.^15^ Though a few mechanisms were suggested by the authors, the small experimental dataset limited their conclusiveness, thus highlighting the need for a more systematic study. More recently, the growth process has been explained using supersaturation of the growth units as an important parameter to control the exposed crystal faces of the NPs, thereby affecting their shape.^17^

Investigating the growth kinetics of anisotropic NPs would lead to a better understanding of the shape evolution. The kinetics of a silver-mediated Au NR system was studied by Bullen *et al.* using UV-visible spectrophotometry, and they report the growth rate to be dependent on the concentration of the reducing ascorbate monoanion and pH around the p*K*_a_ of AsA.^18^ A few other studies examining the growth kinetics of Au NRs include the use of small-angle X-ray scattering (SAXS)^19^ and atomic force microscopy coupled with electron microscopy of surface-bound NPs.^20^ However, the datasets, being relatively small, are not representative of the studied sample space, thereby preventing statistically significant analysis of growth and the associated kinetics. Additionally, there is no report on understanding the growth kinetics for Au NPs of different shapes, as most of these studies primarily focus on Au NRs. On the contrary, various hypotheses have been reported in the literature to explain the possible mechanisms that lead to the growth of anisotropic NRs from the initial spherical gold seeds.^21–23^ A recent investigation by Edgar *et al.* suggested a stochastic growth mechanism for Au NRs, where different seeds grow non-uniformly to attain the final NR shape.^24^ Performing statistical studies for identifying the most critical parameters in NP formation could provide precise control of Au NP surface chemistry, thereby giving better yields. A recent study reported by the Murphy group used a fractional factorial design of experiments to understand the important experimental variables and quantify their effects when synthesizing Au NRs.^25^ However, the study was limited only to Au NRs.

The primary focus in seeded growth is the use of the single surfactant CTAB and all the above studies have shed light on understanding shape control, kinetics and/or mechanistic insights for such a process. The presence of an additional co-surfactant in seed-mediated growth has been reported to significantly improve the dimensional tunability. The effect of binary surfactant mixtures of CTAB with oleyl functional groups such as sodium oleate (NaOL), sodium linoleate and oleylamine on the shape tunability of Au NRs was investigated by Ye *et al.*^26^ A further extension of this work, studying the overgrowth of Au NRs, by Khlebtsov *et al.* also reported dimensional and plasmonic tunability of Au NRs using binary surfactant mixtures of CTAB and NaOL.^27^ Previous work carried out by our group demonstrated shape tunable synthesis of anisotropic Au NPs through the use of binary surfactant mixtures of didodecyldimethylammonium bromide (DDAB) and CTAB.^28^ However, the scope of these studies was limited to changes in a few synthesis variables and they did not delve into the growth mechanism or kinetics of growth. To our knowledge there are no reports of a systematic study investigating the growth and growth kinetics of different anisotropic shapes of Au NPs involving binary surfactant mixtures. An additional challenge while employing a secondary surfactant is an increase of one more reaction parameter that can be varied to control the growth in addition to the studied parameters as in the case of a single surfactant system. This calls for better visualization of the data space in order to map the morphological transitions from one shape to another. An effective tool to accomplish this is a ternary representation which has been commonly used in other research domains.

Here, we report for the first time a ternary representation of the data space for a compact visualization of shape transitions of Au NPs as a function of reaction parameters. We also study and report for the first time the growth and growth kinetics of different anisotropic shapes of Au NPs by varying the pH, amount of the reducing agent, and seed type among other factors in seed-mediated growth employing binary surfactant mixtures of CTAB and oleic acid (OA). Multivariate linear regression analysis is performed on the experimental dataset to determine statistically significant variables for shape control of Au NPs. The kinetics studies coupled with the statistical analysis enable us to understand and provide insights into the growth mechanism that is postulated from a classical nucleation standpoint as a function of supersaturation of growth units.

## Experimental section

Cetyltrimethylammonium bromide (CTAB, 99%+) was bought from Acros Organics and d-(−)-isoascorbic acid (AsA, >99%) was purchased from Fluka. Silver nitrate (AgNO_3_), gold(iii) chloride trihydrate (HAuCl_4_·3H_2_O, 99.999%), oleic acid (OA) and sodium borohydride (NaBH_4_, ≥98%) were purchased from Sigma-Aldrich. Sodium citrate dihydrate (Na-citrate, ACS grade from Merck), hydrochloric acid (HCl, 37% fuming from Merck Millipore) and sodium hydroxide (pellets AnalaR NORMAPUR® ACS) were purchased from VWR. All chemicals were used as received without further purification. Distilled deionized water (resistivity ∼ 18.2 μΩ cm, pH ∼ 6.5) purified using a Millipore water purification system (MQ water) was used for the synthesis and characterization of Au NPs.

A wide array of anisotropic Au NPs has been synthesized in this study employing the seed-mediated growth strategy. Herein, two different Au seeds – CTAB coated and Na-citrate coated seeds – have been used to investigate the growth of anisotropic Au NPs synthesized *via* single and double seeding as outlined below. Characterization of the anisotropic Au NPs, kinetic studies to investigate their growth mechanisms and statistical analysis to understand and evaluate trends in data sets were carried out and are discussed in the subsequent sections.

### Synthesis of Au seeds

Two types of Au seeds – CTAB coated and citrate coated – were synthesized.

### Synthesis of CTAB coated gold seeds

CTAB coated Au seeds were prepared by reduction of Au^3+^ ions in solution with the aid of a strong reducing agent, NaBH_4_, following the synthesis protocol reported previously by our group.^28^ Briefly, 5 ml of a 0.2 M CTAB solution was mixed with a 0.4 mM HAuCl_4_ solution, to which 1.6 ml of freshly prepared 3.75 mM NaBH_4_ was added. The mixture was reacted for 2 min under vigorous stirring. Thereafter, the seeds were aged for 30 minutes at room temperature in order to remove any gas formed during the reaction.

### Synthesis of citrate coated gold seeds

Citrate coated gold seeds were prepared following the method adapted from the work of Gole A. *et al.*^29^ In essence, 0.5 ml of 10 mM Na-citrate was mixed with 0.5 ml of a 10 mM HAuCl_4_ solution. 18.4 ml of MQ water was added to this mixture, followed by the addition of 0.6 ml of an ice-cold solution of 0.1 M NaBH_4_ under vigorous stirring. The mixture was reacted for 3 minutes and subsequently aged for 3 hours at room temperature. This was carried out to allow excess borohydride to be decomposed by water.

The CTAB or citrate coated seeds were used for the growth of anisotropic NPs without further purification.

### Synthesis of anisotropic Au NPs

**Fig. 1:**
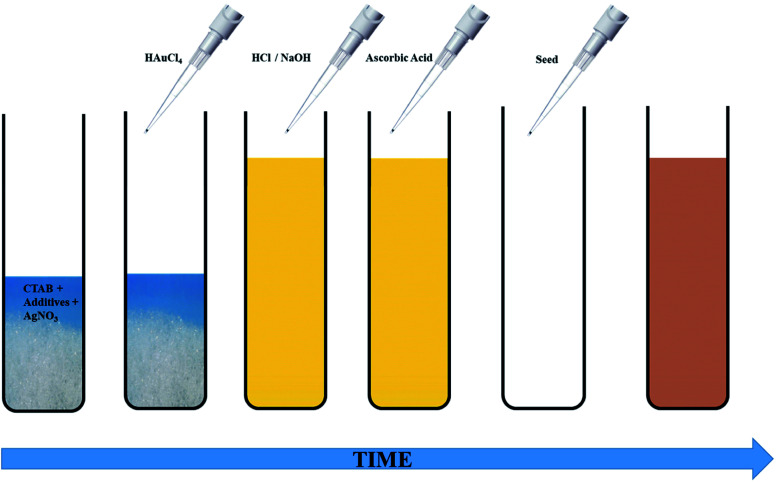
Schematic representation of the Ag-assisted seeded growth of Au NPs.

The seed-mediated growth protocol for the synthesis of anisotropic Au NPs was adapted from our previous work.^30^ The growth solution consisted of CTAB, HAuCl_4_, varying amounts of AgNO_3_, AsA and in some cases, OA. The pH of the growth solution was adjusted before addition of AsA. CTAB or citrate coated spherical Au seeds were added to the growth solution to obtain anisotropic NPs of different shapes and sizes. Two different seeding strategies were studied, namely single seeding and double seeding. For both the cases, the growth solutions were prepared in an identical fashion; however, the seed solutions used for initiating anisotropic growth were different. A typical schematic of this protocol is shown in Fig. 1.

The experimental process for single-seeded growth is described herein. 1.2 g of CTAB and varying amounts of the co-surfactant, OA (Table S1†), were dissolved in 15 ml of MQ water (80 °C) *via* vigorous stirring (1200 rpm). After the solution turned transparent, the temperature was lowered to 35 °C. Thereafter, 750 μl of a freshly prepared 4 mM AgNO_3_ solution was added to the surfactant mixture and stirred for 15 min at 35 °C. This was followed by addition of 15 ml of 0.86 mM HAuCl_4_·3H_2_O and the solution was further stirred for 15 min at the same temperature. If required, the pH of the solution was adjusted at this stage using different volumes of either 1 M HCl or 0.1/0.01 M NaOH. Thereafter, different volumes of 128 mM AsA (Table S1†) were added to the reaction mixture followed by stirring at 1000 rpm to obtain a colourless solution. The disappearance of the colour indicates the reduction of Au^3+^ to Au^1+^.^13,28^ For the single-seeded experiments, 96 μl of the Au seed solution (CTAB or citrate coated) was finally added to the growth solution under vigorous stirring (1200 rpm). In the case of double seeded experiments, an intermediate growth solution was prepared using the protocol outlined above (with 30 μl OA and identical steps afterwards), ending with the addition of 96 μl of the Au seeds (CTAB coated). The name double-seeded is coined based on the fact that after 30 s, 300 μl of this intermediate growth solution was used as a seed for further growth in fresh growth solutions.

For both single and double-seeded experiments, after 30 s of the addition of the seeds into the growth solution, stirring was stopped, and the resultant reaction mixture was left for 8 h at 35 °C for completion of the reaction. Thereafter, the reaction mixture was centrifuged twice at 11 000 rpm for 20 min and the Au NPs were re-dispersed in 5 ml of MQ water.

Detailed information about the experimental conditions used for the synthesis of Au NPs is reported in Table S1.†

### Characterization of Au NPs

A Hitachi S-5500 Scanning Transmission Electron Microscope (STEM) was used for studying the sizes and shapes of the NPs. Bright-field STEM (BF-STEM) images were acquired at an accelerating voltage of 30 kV. TEM grids were prepared by placing a few drops of the sample on a Formvar carbon-coated 300 mesh copper grid (Electron Microscopy Sciences) and wiping immediately with Kimberly-Clark kimwipes to prevent further aggregation owing to evaporation at room temperature. Imaging software SPOTCAM 4.0.1 was used for measuring particle sizes, in which the image pixels were calibrated against the scale bar of the image.

UV-Vis spectra for studying the growth kinetics and obtaining the plasmonic peaks of the NPs were acquired using a UV-2401PC (Shimadzu) spectrophotometer. The spectra were collected over a spectral range from 400–900 nm.

### Study of growth kinetics

The growth of the Au NPs was tracked over time by obtaining spectra of the growing NPs over the range 400–900 nm using a Shimadzu spectrophotometer (UV-2401PC). The protocol followed was adapted from the work of Bullen *et al.* in which the authors studied the overgrowth of Au NPs.^18^

In the current study, 3.5 ml of the growth solution was immediately withdrawn after addition of seeds and transferred into a UV-Vis cuvette (disposable cuvette from PLASTIBRAND®). The instrument was also maintained under the reaction conditions (35 °C). The time difference from sample withdrawal until the start of spectral acquisition was noted to be approximately 30 s for each experiment. This time lag in acquiring spectra was compensated for during analysis of the kinetic data. UV-Vis spectra in the range 400–900 nm were obtained for 2.5–6 hours (longer time for lower pH samples) by using the UV-Vis repeat mode, in which the instrument continuously measures absorbance over time.

The UV-Vis instrument can measure the absorbance of metallic Au^0^ that is formed upon addition of seeds due to an autocatalysis reaction involving Au^1+^ following first-order kinetics.^18,24^ The area under the spectrum gives a measure of the total quantity of Au^0^ formed from the available Au^1+^ in the solution upon addition of seeds. With time, the areas under the curves increase monotonically until they are saturated. Individual areas obtained for a single type of NP were normalized against the area of its final spectrum obtained at the end of UV-Vis measurements for ease of quantitative analysis. The curve between the normalized area and reaction time was fitted with the sigmoidal 4 parameter equation given below and the first-order rate constant was calculated using the fitting parameters.1
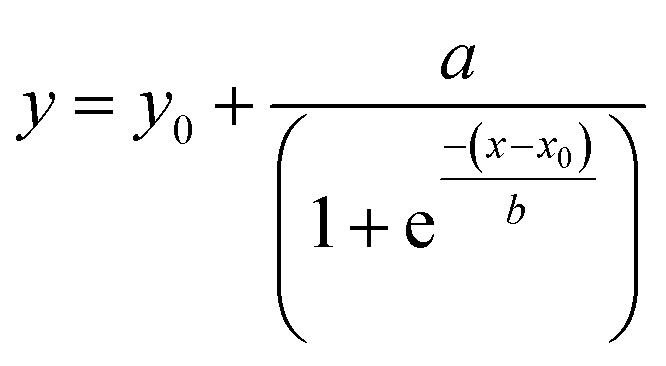


### Statistical analysis

#### Correlation loading plots

Correlation loadings for the multivariate data were performed using Unscrambler X software. The software performs Partial Least Squares Regression (PLSR) in order to explain the relationship between the input and output data. The experimental conditions like the amount of AsA, OA, *etc.* were used as input variables while the shape of NPs obtained, localized surface plasmon resonance (LSPR) peak position, *etc.* were treated as output variables. The data were normalized and cross-validated and then PLSR was performed. The regression model obtained gives information about similarities and differences in the sample dataset. The correlation loadings explaining the positive and negative correlations among the variables can be visualized through score plots (Fig. S10–S12†). Based on the formulated model, the plot indicates the variables in the dataset that are responsible for 100% and 50% explained variance. The results from these models were compared against experimental observations for a statistical understanding of the system parameters.

### Multivariate linear regression analysis

JMP® statistical discovery software from SAS was used to perform multivariate linear regression analysis on the experimental data. In essence, variables such as pH and volumes of OA, AgNO_3_, AsA and the seed were used to obtain the predicted values of parameters such as rate constant, LSPR peak wavelength, number of LSPR peaks, long axes of the Au NPs and their AR. The model was constructed to map the effect of different reaction parameters on the shape and properties of the resultant NPs by performing backward selections of the various predictors/variables. The effect of the various predictors on the variability of the response parameter was analyzed by checking the *R*^2^ values of the fits. The least important covariates (with high prob > *t* values) were omitted in each successive run of the model in order to obtain the predictors that provide significant information on the variability of the response parameter. The predicted values of response parameters acquired from the final model were then compared with the actual values obtained.

## Results

Seeded growth synthesis of anisotropic Au NPs has garnered remarkable interest owing to the wide design space and well-defined control of parameters to influence the shape, size, yield, and morphology among others of the final products. The focus has been on understanding the growth of Au NRs synthesized using a single surfactant, CTAB, and by varying other parameters, such as the reducing agent, metal precursor, surfactant concentration, seed concentration, additives and so on. However, the interplay of different reaction parameters makes the mechanistic understanding of the growth process a complicated topic. For obtaining other anisotropic shapes (other than NRs), addition of a second surfactant molecule has been shown to be effective. This further expands the set of tunable parameters and, therefore, for controlling the growth, a visual representation of the design space becomes an obvious necessity. In this regard, we believe that our ternary space representation to map the different morphologies obtained by the seeded growth methods is a unique methodology being reported for the first time here.

**Fig. 2:**
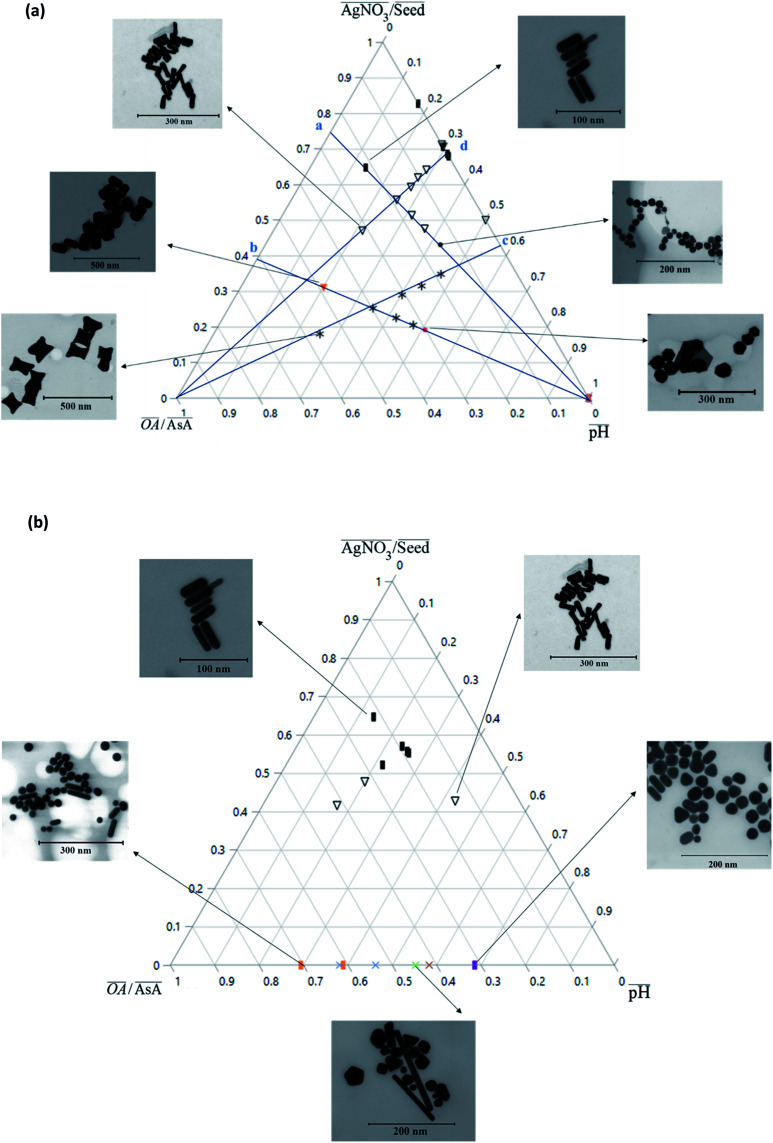
(a) Ternary plot showing the different shapes of Au NPs obtained under different synthesis conditions. (b) Ternary plot showing the different shapes of Au NPs obtained under different synthesis conditions for OA = 0.

Fig. 2(a) shows an overview of the different shapes of Au NPs that were obtained under the different synthesis conditions employed in the current study. We report for the first time a ternary space representation as a tool for showing morphological transitions of anisotropic Au NPs as a function of reaction parameters. The ternary space diagram shows the variation of the shapes as a function of three normalized parameters – (i) the volume ratio of OA to AsA 
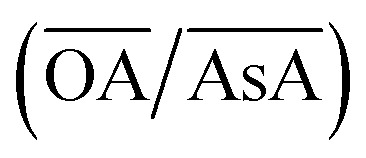
, (ii) the volume ratio of silver nitrate to CTAB coated seeds 
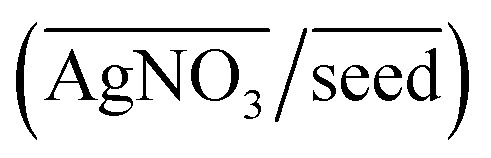
 and (iii) the scaled pH of the solution after addition of AsA 
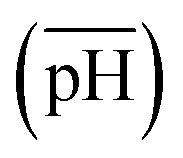
. The synthesis parameters are grouped based on the fact that both OA and AsA play the role of reducing agents, while AgNO_3_ and seeds are known to play important roles in determining anisotropic growth. It should, however, be noted that each of the above parameters has been normalized to its respective highest value followed by utilization of ternary transform in SigmaPlot that normalizes the data to a ternary space for the sake of better visualization of morphological transitions. A first glimpse at the ternary space shows that a large number of shapes can be synthesized ranging from spheres to NRs, etched nanorods (ERs), tetrahexahedra (THH), multifaceted structures, and the recently reported nano*makura* (NM).^30^ Another important reason to convert the dataset into a ternary space diagram is the ease with which the varying parameters can be studied to account for the morphological changes in the obtained shapes along different connecting lines. This shall enable a qualitative understanding of the role of various synthetic parameters in affecting the shape transitions of Au NPs. Thus, the ternary space plots shown here only provide a visual means to group and classify various morphologies as a function of reaction parameters, as opposed to representing thermodynamical phase equilibria as in ternary phase plots.

In order to understand the effect of varying parameters on shapes, a set of connecting lines (a, b, c and d) are used in Fig. 2(a). Along line ‘a’, as the pH increases, the morphology of the Au NPs changes from NR → ER → sphere. For another line ‘b’, if the pH increases, the shapes change as follows: THH → NM → multifaceted sphere. Both lines show that an increase in pH of the reaction medium results in the formation of structures with an increasing number of facets. An increase in pH can be equated to an increase in the strength of the primary reducing agent (AsA) affecting the overall growth kinetics. On the other hand, an increasing 
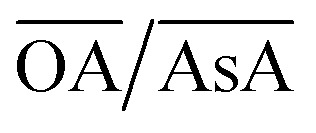
 does not change the morphology of the Au NPs, represented by the line ‘c’. This line shows that NM is formed as the only morphology under these conditions. However, the line ‘d’ represents the fact that increasing 
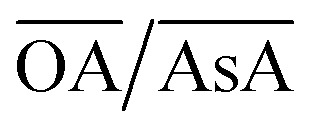
 changes the morphology from NR → ER. Thus, an interesting observation from lines ‘c’ and ‘d’ is the fact that OA or AsA has a comparatively lower effect on the morphology of the obtained NPs when the pH is appropriately tuned. Overall, pH seems to have a dominating role in controlling the growth of these NPs. Interestingly, a large number of points coincide on the scaled 
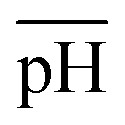
 vertex of the ternary plot. In order to have a closer look at the morphologies involved, a separate ternary plot is drawn in Fig. 2(b) for OA = 0.

As can be seen from Fig. 2(b), even without the use of OA, it is possible to synthesize a plethora of shapes ranging from NSs, NRs, and ERs to multifaceted structures. However, there are clear zones in the plot where NRs and ERs lie, indicating that appropriate tuning of the synthesis parameters is an essential aspect for shape control. On the other hand, the absence of AgNO_3_ (represented by the base of the ternary plot opposite the 
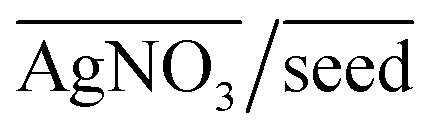
 vertex) hampers the selectivity of one shape over other shapes in the final product. However, this selectivity can be tuned by adjusting AsA and pH. An increase in pH coupled with a decrease in AsA leads to the formation of longer NRs and spheres *via* several other shapes that include prisms and multifaceted structures.

In a typical synthesis protocol (without the use of a co-surfactant), the yellow coloured growth solution turns colourless upon addition of the primary reducing agent, AsA. This is an indication of the complete conversion of Au^3+^ to Au^1+^. In the case of our experiments, the pH of the growth solution was adjusted before the addition of AsA *via* using NaOH or HCl. The pH values reported were measured after the addition of AsA and before the addition of the seed. It is worth mentioning that the growth solution decolourized even before the addition of AsA when OA was present in the reaction mixture. This happened more noticeably when increasing the pH, pointing once again to the fact that pH is a significant variable for growth control.

### Effect of pH on the shape

**Fig. 3:**
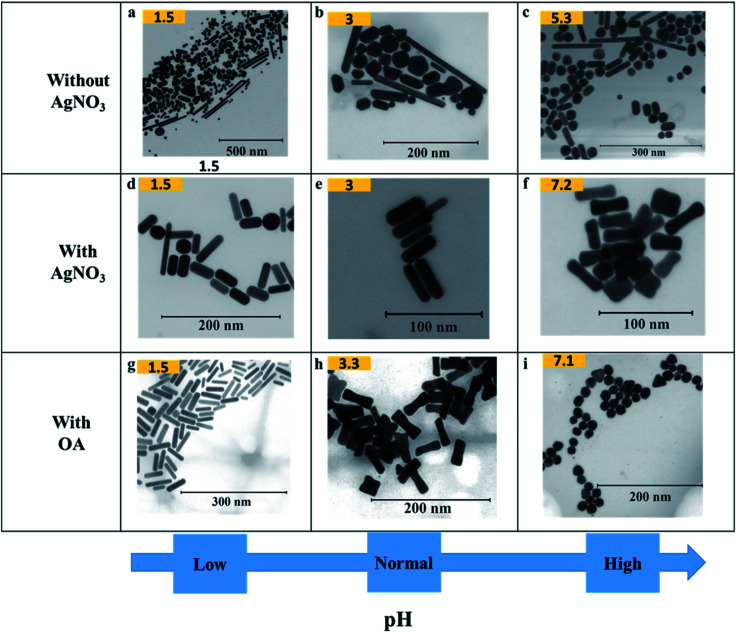
Effect of pH on the shape of Au NPs. (a–i) Representative S(T)EM images of different Au NPs (pH after adding AsA).

Fig. 3 shows how pH affects the shapes of Au NPs. The pH of the growth solution was measured and changed to a predefined pH (in the range 1.5–10) by addition of HCl or NaOH (Table S1†). The pH values reported in the figure above were measured after addition of AsA. In a typical experiment (without the addition of HCl or NaOH), the pH of the growth solution after addition of AsA was measured to be around 3. In such a case, the absence of AgNO_3_ results in no well-defined shape selectivity (Fig. 3(b)). However, in the presence of AgNO_3_ (pH ∼ 3), monodisperse NRs with an AR of 3.2 ± 1.0 (*L* = 40 ± 8 nm, *D* = 14 ± 4 nm, Fig. 3(e)) were obtained. The role of AgNO_3_ has been investigated by us and several other researchers in inducing anisotropy in seed-mediated growth.^14,28^ Concomitant addition of OA results in ERs with an AR of 2.9 ± 0.9 (*L* = 44 ± 6 nm, *D* = 17 ± 6 nm, Fig. 3(h)).

In the absence of AgNO_3_, increasing the pH results in better shape selectivity (compare Fig. 3(a) and (c)). Upon addition of AgNO_3_, improved shape selectivities can be observed under both low and high pH conditions. At low pH (∼1.5), addition of AgNO_3_ leads predominantly to NRs with an AR of 3.2 ± 2.0 (*L* = 42 ± 13 nm, *D* = 16 ± 6 nm, Fig. 3(d)). Smoother and higher AR NRs (3.9 ± 1.2, Fig. 3(g)) are obtained upon addition of OA. At high pH (∼7), addition of AgNO_3_ leads predominantly to ERs with an AR of 2.8 ± 1.0 (*L* = 36 ± 7 nm, *D* = 15 ± 5 nm, Fig. 3(f)). Addition of OA leads to the formation of spheres of size 18 ± 3 nm (Fig. 3(i)).

At low pH (∼1.5), in the presence of OA, NRs are obtained as discussed above. A similar morphology can be observed in the case of pH 3 in the absence of OA (Fig. 3(e)). When one compares Fig. 3(h) and (f), similar morphologies, *i.e.* ERs can be observed. Therefore, by adjusting either the pH or the amount of OA, finer control over the morphology of the obtained Au NPs can be exerted.

An increase in pH, however, promotes shape selectivity, resulting in more spheres and NRs, in comparison to other shapes (Fig. 3(c)). However, in the absence of AgNO_3_, a certain degree of shape selectivity can be achieved. As the pH increases, the standard deviation of the long axis of the NPs goes down from 75 nm to 24 nm as the number of different/irregular shapes obtained decreases. In the presence of AgNO_3_, shape selectivity is achieved, and uniform anisotropic NPs are obtained. At low pH, NRs of different sizes are obtained, and uniform NRs are obtained when the pH is unaltered (pH = 3). As the pH increases, the size of NRs decreases and at pH 7.2, the shapes change to ERs.

Two different seeding techniques have been employed – single seeding and double seeding. In the latter case, intermediate growth solutions were added as seeds to fresh growth solutions. Our group were the first to report the shape nanomakura obtained under such conditions.^30^ However, when the pH is varied, a transition from THH NPs to multifaceted sphere-like NPs is observed (Fig. S9†). This trend of shape transition is similar to the trend observed for single seeding with OA where a shape transition occurs from NRs to ERs to spheres under similar conditions. pH also affects the reducing capability of AsA, which is then expected to exert an influence on the morphologies obtained.

### Effect of AsA on the shape

**Fig. 4:**
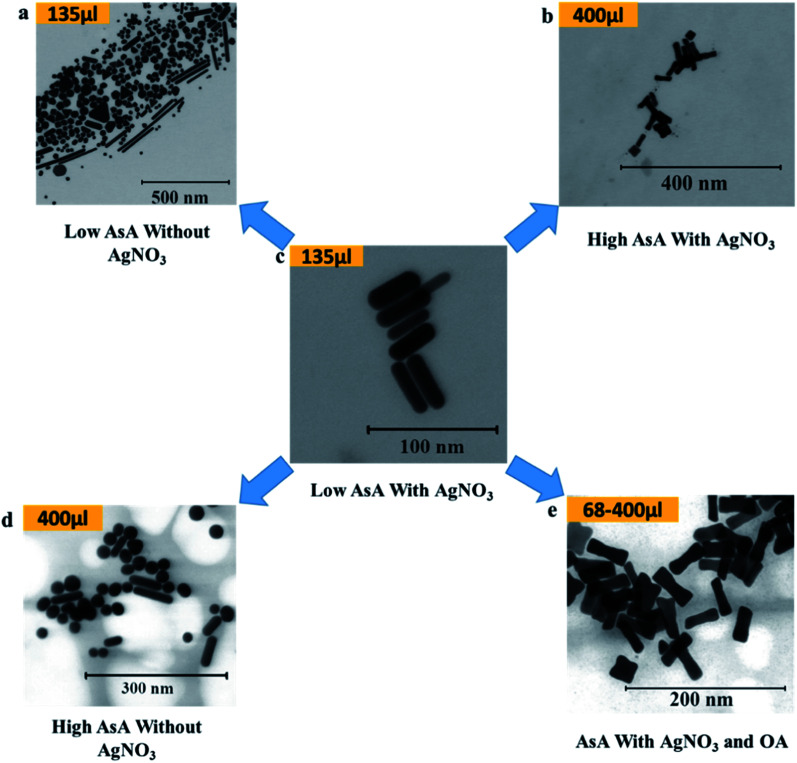
Effect of ascorbic acid on the shape of Au NPs. (a–e) Representative S(T)EM images of different Au NPs.

AsA was varied between 68 μl and 400 μl in order to study its effect on the shapes of the synthesized Au NPs. Fig. 4 shows the different shapes that were obtained on varying AsA with and without using AgNO_3_ and OA. The lowest volume of AsA used in the study set produced no Au NPs in the absence of OA. However, in the presence of OA under these conditions, ERs were obtained. In essence, ERs of similar AR (2.8 ± 0.2) were also obtained when the amount of AsA was increased keeping the amount of OA unchanged (Fig. 4(e)). Under these conditions, there is no appreciable effect of the quantity of AsA on the shape and AR of ERs.

Conversely, in the absence of AgNO_3_ and OA (Fig. 4(a) and (d)), different shapes were obtained. However, the shape selectivity of NRs and spheres over other shapes improved on increasing the amount of AsA. In the presence of AgNO_3_, at 135 μl of AsA (Fig. 4(c)), uniform NRs (AR 3.2 ± 1, length 40.2 ± 7.7 nm) were obtained. When the amount of AsA is increased, the obtained shapes changed from NRs to ERs – a representative ER (AR 3.0 ± 0.8, length 39.4 ± 6.1 nm) at the highest amount of AsA is shown in Fig. 4(b).

In order to understand semi-quantitatively the influence of reaction parameters on the shapes of the obtained Au NPs, the growth of the Au NPs was followed using UV-Vis spectrophotometry.

### Growth kinetics of NPs

**Fig. 5:**
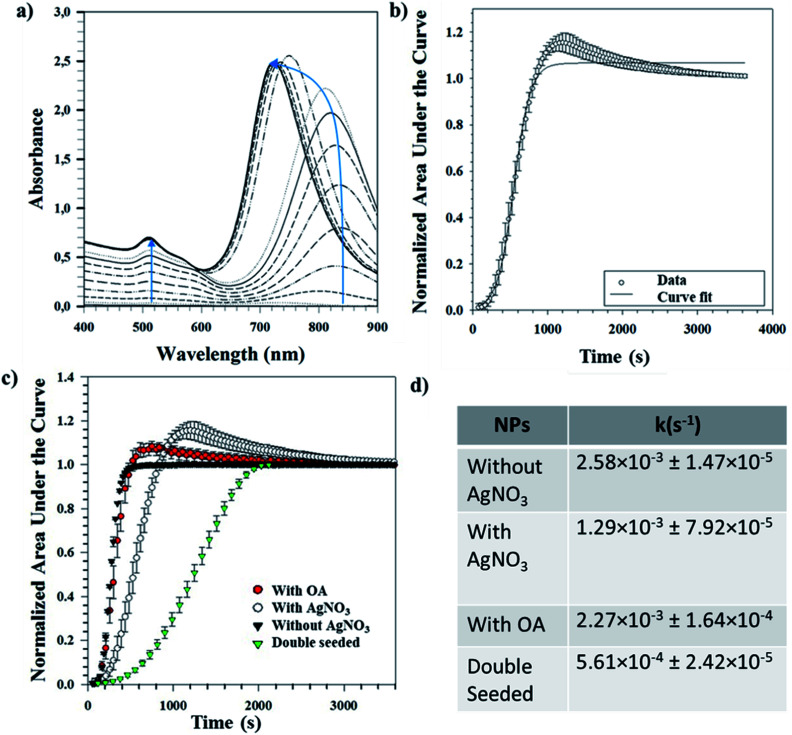
(a) Tracking the growth of Au NRs using UV-Vis spectrophotometry; (b) growth curve depicting the normalized areas and corresponding sigmoidal fit; (c) growth curves obtained for different synthesis conditions of Au NPs; (d) table showing the first-order rate constant for different synthesis conditions.

Tracking the growth kinetics helps us to understand how the Au seeds grow into the final anisotropic shapes. To our knowledge, we report here for the first time a reliable, simple and robust spectroscopic method capable of tracking the growth of different shapes of Au NPs. Based on this novel UV-Vis method, the temporal evolution of the absorbance values was noted using UV-Vis spectrophotometry for a small sample volume of the reaction mixture withdrawn immediately after the addition of seeds. The LSPR peak(s) can be spotted almost immediately after the start of the measurements and tends to grow as a function of time. Fig. 5(a) shows a representative UV-Vis spectrum for NRs obtained over time. It is worth mentioning that the LSPR maximum for the longitudinal peak first undergoes a bathochromic shift followed by a hypsochromic shift (indicated by the blue line in Fig. 5(a)). For the same NRs, the area under each spectral curve was thereafter calculated, normalized and plotted against time to which a 4-parameter sigmoidal curve was fitted (Table S3†), as shown in Fig. 5(b). The first-order rate constant *k* was calculated from the fitting parameters and gives an idea of the growth rates of the NRs.

The same procedure was followed for different reaction conditions such as with and without AgNO_3_, with OA and double seeding which yield random shapes, NRs, ERs and NMs, respectively. Fig. 5(c) shows the normalized areas under the curves obtained for these different conditions. High values (∼1) of regression coefficients show that the fitted curves are a statistical representation of the growth kinetics. The sigmoidal nature of the curve in all the above cases indicates that the reaction is autocatalytic for all the studied conditions. Similar autocatalytic growth of NRs has been reported previously.^24,25,31^ There is an initial slow growth phase, characteristic of sigmoidal growth, for all the shapes; however, the exponential growth phase starts at increasing time points for samples without AgNO_3_ followed by samples with OA, further followed by samples with AgNO_3_ and finally double seeded samples. These time points are in coherence with the time points at which a visible colour change occurs (for all samples). Our observation is in line with previous reports, where the role of AgNO_3_ in retarding growth kinetics is well established.^18,23^ Further, we observe that the addition of OA shortens the growth lag, indicating the role of OA as a reducing agent, as discussed before. In the case of the double seeded experiments, the amount of OA used is higher; however, there is retarded growth kinetics in comparison to the other shapes. As reported previously by our group, the seeds, in this case, are not homogeneous (different shapes),^30^ which can explain the presence of different shapes in the growing media, further supported by broad UV-Vis peaks that continue to grow temporally. Therefore, the spectral signatures of individual axes are masked by the broad shape distribution present at all times.

A single UV-Vis run for the whole wavelength range (400–900 nm) takes ∼90–100 seconds resulting in the loss of temporal data points leading to a loss of kinetic information in the initial phase of the reaction. To verify the effect of the increased number of time points, UV-Vis measurements were performed for smaller wavelength ranges for NRs and ERs. The fitting parameters obtained for the longitudinal and transverse axes in our representative datasets were comparable to the fitting parameters obtained for the whole wavelength range (400–900 nm) (Table S4†). In addition, the fitting parameter representative of kinetics (*b* from eqn (1)) of the longitudinal axis represents the fitting parameter (*b* from eqn (1)) obtained for the whole wavelength range. Therefore, once the locations of the LSPR peaks of the desired shape are known, a smaller range wavelength scan around the LSPR will be advantageous to facilitate data collection for an increased number of time points. This will lead to higher accuracy in the capture of the kinetics of faster-growing shapes, especially in the initial growth phase. Further, since we have shown that small range scans provide representative fitting parameters for the overall growth kinetics, these can be used for understanding the overall growth kinetics.

The first-order rate constants (*k* values) obtained for these conditions are shown as a part of Fig. 5(d). These complement the ongoing discussion about the role of AgNO_3_ and OA in kinetics, where their addition retards the overall kinetics. The experiments with AgNO_3_ show slower kinetics than the ones with OA. The reported *k* values are obtained averages from three different repeats for each condition. The low standard deviations indicate the reproducibility of this method for determining growth kinetics of anisotropic shapes. Since this method uses UV-Vis spectrophotometry and we have shown applicability for different shapes, we believe our method can be expanded to follow the growth of other shapes yielding information about the growth kinetics.

## Discussion

Tuning the growth of Au NPs is controlled by the interplay of different synthetic parameters such as the nature of the surfactant or co-surfactant, pH, counter-ions, type of seed, chemical reagents and others. Besides the roles that these factors play in influencing the growth, one of the pertinent factors frequently overlooked in the field of seeded growth methods for Au NPs is the shelf-life of chemical reagents. In a recent study, Scarabelli *et al.*^32^ showed that a shelf life of one week for AgNO_3_ and AsA is necessary for the replicable synthesis of NRs (*i.e.* NRs with the same AR and LSPR peaks). The study further recommended that chemicals such as AsA and AgNO_3_ should be stored at 4 °C and HAuCl_4_ should be refrigerated. Counting on our previous experience in shape control of anisotropic structures using a seeded growth protocol, we investigated the shelf-life of the reagents, besides other reaction parameters, to gain a statistical understanding of the variability in obtained data. Our findings show that the growth kinetics of the NPs are largely governed by the shelf-life of the chemicals. Although the same shapes can be obtained while following the same recipe, a high standard deviation in determined growth rate constants can be observed when AgNO_3_ and AsA are more than a day old. For instance, the standard deviation in the case of the determined growth rate constants for Au NRs increases by one order of magnitude for the experiments in which freshly prepared chemicals were used (chemicals used on the same day as prepared) in comparison to the experiments in which the chemicals had been stored several days before use (Table S5†).

Correlation loadings (Fig. S10–S12†) using the partial least squares method show the statistical importance of different reaction parameters for shape tunability. AgNO_3_ is shown to be statistically significant in influencing shape selectivity. Further, the need for OA to obtain ERs when parameters such as pH and amount of AsA are kept constant is also evident. Our findings strongly suggest that for better control of kinetics and for fine-tuning of shapes in a seeded growth method employing binary surfactant mixtures, a one-day shelf life is recommended for AsA and AgNO_3_.

Therefore, in designing our experimental matrix, where we investigate the effects of pH, AsA, OA, type of seed and other reaction parameters, we have used the guidelines for chemicals as indicated by our correlation loadings study. The synthesis of Au NPs using a seeded growth method requires a complex mixture of both thermodynamic and kinetic controls, resulting in a large number of parameters to be studied. This necessitates a multivariate approach to understand their roles in controlling the growth kinetics leading to different shapes.

Here, we report for the first time that the presence of OA in the growth medium decreases the overall growth rate showing that OA has a retarding role in controlling the growth kinetics. Previous reports have shown that the presence of AgNO_3_ in the growth medium slows down the kinetics.^18^ Our findings in growth kinetics determined using the UV-Vis methodology also reflect the same. On the other hand, the overall growth rates increase when the pH of the growth medium or amount of AsA is increased. Among other factors, the growth rate is influenced largely by the reduction rate of Au^1+^ (reduction product of Au^3+^, the reduction achieved in the synthesis process using AsA) to Au^0^, which is dependent on the reduction environment. An increase in the reduction rate can be achieved either by increasing the reduction potential or by increasing the amount of the reducing agent used. At a higher volume of AsA, there is a higher availability of the reducing agent, while an increase in pH above the p*K*_a_ value of AsA (p*K*_a_ = 4.7) increases the reduction potential of AsA. These effects, in turn, increase the overall growth rates represented by the increase in the *k* values. Although the change in pH or AsA results in similar effects, it is important to emphasize the dominant role of pH in controlling growth rates besides the formation of different shapes, as depicted in ternary space representation. In the case of the effect of pH in the presence of OA, the growth rate constants increase by one order of magnitude when a high pH is maintained. Under similar conditions, different shapes *viz.* NRs and sphere-like NPs are obtained, as inferred from their LSPR signatures at 761 nm and 520 nm, respectively.

With an aim to statistically interpret the variability in growth kinetics as a function of the reaction parameters, multivariate analysis was performed using JMP to evaluate the interactions among reaction parameters such as pH, amount of AsA, amount of OA, *etc.* and output response parameters such as physico-chemical properties of Au NPs (like length of the long axis (*L*), AR, *etc.*) and the overall growth rate constant. The equation to predict the output response parameter obtained using linear multivariate regression has the following form.*y*_i_ = *a*_1_*x*_1_ + *a*_2_*x*_2_ + … + *a*_*n*_*x*_*n*_ + *C*_i_where *y* is the predicted parameter that depends on predictor variables (*x*_1_, *x*_2_…) and the constant *C*_i_ is the intercept of the equation. A summary of the predictor equations is presented in Table 1. The detailed multivariate analysis and the correlation loadings of all the studied parameters are provided in Fig. S10–S13.†

**Table 1:** Table showing the parameters obtained through multivariate linear regression

*y*	*x*	*a*	*c*
*k*	LSPR	−1.65 × 10^−5^	8.13 × 10^−3^
	No. of LSPR peaks	1.12 × 10^−3^	
	AsA	4.73 × 10^−6^	
	pH	4.87 × 10^−4^	
*L*	AgNO_3_	−6.19 × 10^−2^	5.68 × 10^1^
	*k*	−1.31 × 10^4^	
	LSPR	2.23 × 10^−1^	
	AR	−3.78 × 10^1^	
LSPR	OA	0.127 × 10^1^	2.83 × 10^2^
	AgNO_3_	1.07 × 10^1^	
	AsA	1.30 × 10^−1^	
	No. of LSPR peaks	4.59 × 10^1^	
	*L*	9.00 × 10^−1^	
	AR	5.61 × 10^1^	
No. of LSPR peaks	*k*	1.62 × 10^2^	−0.35 × 10^1^
	LSPR	7.76 × 10^−3^	
AR	OA	−2.44 × 10^−2^	8.24 × 10^−1^
	AsA	−1.54 × 10^−3^	
	pH	−2.10 × 10^−1^	
	LSPR	5.30 × 10^−3^	
	*L*	−1.07 × 10^−2^	

Statistically, *k* can be predicted as a function of LSPR wavelength, number of LSPR peaks, amount of AsA and pH of the growth solution. pH is also observed to be an important predictor for where the LSPR peak will be located. The LSPR peak position is further dependent on the amount of AgNO_3_, which is a statistical representation of the fact that AgNO_3_ plays a dominant role in determining the position of LSPR the peak of the obtained Au NP, thereby affecting shape selectivity, as widely reported in the literature.^14,33,34^ The plasmonic properties of the Au NPs depend on their shape and size (among other factors); therefore, morphological properties of the NPs like *L* and AR are found to statistically influence the LSPR peak position. From the previous discussion, the shapes in turn have been shown to depend on experimental parameters such as AsA, OA, AgNO_3_ and pH. Therefore, the properties of Au NPs like *L* and AR are dependent on each other (which is similar in the case of LSPR with no. of LSPR peaks) and hence appear as a predictor for one another in the multivariate predictor equations.

The multivariate analysis shows that experimental parameters like pH and amount of reducing agent show a strong correlation with the shapes and growth kinetics of NPs; however, in order to understand the growth mechanism involved, an insight into the role of the different chemicals used and their effects on crystal growth is necessary. From our study, we have observed that the experiments with low AsA (68 μl) lead to a complete reduction of Au^3+^ to Au^1+^ after addition of AsA (observable colour change from yellow to colourless of the growth solution), but we observe no growth of the Au seeds under these conditions. This indicates a need for excess AsA for reducing Au^1+^ to Au^0^ in the presence of the Au seeds. When the amount of (excess) AsA increases, an increase in the overall growth rate is observed owing to a higher amount of Au^0^ produced *via* reduction of Au^1+^. The *k* values obtained indicate a similar increasing trend, for example, the *k* values of experiments with AgNO_3_ vary from 0 for 68 μl of AsA to 1.94 × 10^−3^ s^−1^ at 400 μl of AsA. Interestingly, in experiments with low AsA (68 μl) (which does not result in growth of Au seeds otherwise), ERs were observed, although with slow kinetics (*k* = 1.48 × 10^−3^ s^−1^). This happens because OA also behaves as a reducing agent, owing to the C–C double bond that provides electron density for reduction.^35,36^ The importance of OA as a reducing agent is further verified through observations in experiments performed under high pH conditions. A colour change from yellow to colourless (of the growth solution), indicating the formation of Au^1+^ was observed (upon addition of OA) even before the addition of AsA, the primary reducing agent. OA has a p*K*_a_ of 5.02 and in the case with high pH, the reduction potential of OA is thus expected to increase, leading to the formation of Au^1+^. The total volume of added AsA, under these conditions, is in excess and helps in the formation of Au^0^ and leads to a high *k* value of 6.81 × 10^−3^ s^−1^. It is worth mentioning that under these conditions, sphere-like NPs are obtained. Alternatively, at this pH, OA could have reacted with the NaOH used to adjust the pH and resulted in the formation of sodium oleate, another reducing agent with a p*K*_a_ of 4.99, which was previously shown by Ye *et al.*^26^ Sphere-like particles were also obtained at high pH and in the presence of OA for both single and double seeded methods.

Although shape selectivity can be adjusted by altering the pH or AsA, AgNO_3_ is still an essential requirement to affect the selectivity of one shape over the others. The growth of Au NPs in the surfactant mediated seeded growth method generally follows the following two reduction steps.Au^3+^ + e^−^ = Au^1+^Au^1+^ + e^−^ = Au^0^

In a typical synthesis (without changing the pH), Au^3+^ is reduced using the weak reducing agent AsA resulting in a supersaturated solution of Au^1+^. The second reduction step occurs in the presence of more reductants (excess AsA or secondary reductants) and Au seeds, resulting in the formation of Au^0^ in the solution close to the surface of the seed. The supersaturated solution of Au^0^ around the seed forms the driving force for crystal growth. From a crystallization point of view, the growth of these NPs involves the formation of Au^0^ and integration of the newly formed Au^0^ on the seeds. However, for the formation of Au^0^ in the solution, the Au^1+^ ions have to travel to the surface of the seed where the autocatalytic reaction happens. For a better understanding of the growth, the following steps can be considered schematically.

• Reduction of Au^3+^ to Au^1+^.

• Transport of Au^1+^ to the surface of the seeds.

• Supersaturation of Au^0^ in solution near the surface of the seeds.

• Surface integration of Au^0^.

The change in the growth solution from yellow to colourless was observed for all the experiments and a some AsA or a combination of AsA/OA in the system is spent on this reduction step. Due to this colour change, it is assumed that there is a complete formation of Au^1+^ in all our experiments. A number of mechanisms have been proposed to explain the diffusion of Au^1+^ by different research groups^37–39^ and it is not part of the discussion here. Since our main aim is to understand the shape evolution of Au NPs in a binary surfactant system, we focus our discussion on the last two steps, which are crucial for understanding how the CTAB coated Au seeds develop into different morphologies. Therefore, we believe that growth of the anisotropic Au NPs obtained under different experimental conditions can be explained by considering the supersaturation of Au^0^ in the liquid surrounding the seeds and integration of Au^0^ on the surface of the seed.

The kinetics of this reaction depends on the amount of reducing agent used and its reduction potential. The Au^0^ formed as a result of the reduction step creates a localized supersaturation around the seed; however, the concentration of Au^0^ depends on the amount of AsA available and the pH (indirectly, the reduction potential). At a lower concentration of AsA or low pH, the formation of Au^0^ is slow, leading to lower supersaturation of Au^0^. The formed Au^0^ can therefore easily integrate into crystalline faces with lower energy. This results in the formation of smoother shapes. At high amounts of AsA or high pH, there is an abundance of Au^0^ creating a very high supersaturation and the formed Au^0^ can integrate readily with the seeds. Under these conditions, the formation of Au^0^ is much faster than the integration into the crystal, leading to rough growth.

Extending our mechanism to understand the shapes obtained, we observe that at lower supersaturation, the driving force is less and structures like NRs are formed. These have a smoother surface and well-defined edges. At high supersaturation, rougher NPs like ERs are obtained and at even higher supersaturation values (high pH with OA) an abundance of Au^0^ around the seeds leads to sphere-like shapes. The abundance of Au^0^ is believed to trigger a second nucleation phase, which can lead to higher kinetics and smaller sizes. This can be observed in the first order *k* values determined using our UV-Vis methodology that show an increase by one order of magnitude between NRs and sphere-like structures. In the experiments where the driving force increases, an increase in the first-order rate constant is always observed. This is in synchrony with concepts borrowed from classical crystallization theory where the kinetics of crystal growth depends on the supersaturation and increases as the supersaturation increases. The growth kinetics and shape dependency of NPs on supersaturation can also be extended to the double seeded method, in which case, at low supersaturation, smooth and well-defined tetrahexahedral structures are obtained and at high supersaturation, multifaceted spheres are obtained. This characteristic behaviour between growth and the driving force (supersaturation) can be summarized as follows. Smooth growth occurs at a lower driving force leading to the formation of NRs and THHs while rough growth contributes to the formation of ERs, NMs, spheres, multifaceted spheres, *etc.* These types of growth and crystal structures are in agreement with the classical crystal growth mechanism that describes the effect of supersaturation as a driving force that leads to the formation of smooth and rough structures at low and high supersaturation, respectively. The formation of NRs and THHs is similar to the formation of polyhedral structures; on the other hand, the formation of ERs and sphere-like structures is similar to dendritic and spherulite growth. Thus, by employing an understanding from classical nucleation theory, we believe that our approach will provide further mechanistic insights into the growth of Au NPs in a seeded growth approach employing binary surfactant mixtures.

## Conclusion

Although CTAB mediated seeded growth of Au NPs has been a topic of intensive research for the last decade, the difficulty in visualizing and understanding different interactions during the seed-mediated growth of Au NPs has been a major challenge in understanding the growth mechanisms involved therein. Here, we report for the first time a ternary representation of system parameters to map the morphological transitions of different shapes of Au NPs in the case of binary surfactant mixtures. We also report for the first time the effect of OA on the growth and growth kinetics of Au NPs in such a seeded process. The effect of other reaction parameters such as AsA, pH, and nature of the Au seeds among others on the growth of Au NPs has been studied. A robust method based on UV-Vis spectrophotometry has been developed to determine the growth kinetics of Au NPs of different shapes. Using multiple linear regression, we have studied the correlation among different parameters and identified statistically significant variables that are responsible for shape control. The results from the kinetics study of the different Au NPs in combination with the ternary representations depicting the effect of pH and AsA were used to propose a growth mechanism based on supersaturation to explain the shapes obtained for Au NPs in the presence of binary surfactant mixtures. The proposed mechanism is in line with classical nucleation theory and provides an understanding of the growth of anisotropic NPs in seeded growth processes.

## Conflicts of interest

There are no conflicts to declare.

## Supplementary Material

NA-002-D0NA00214C-s001
