# Supplementary material for: Tuning and tracking the growth of gold nanoparticles synthesized using binary surfactant mixtures
Source: Nanoscale Adv. 2020 Mar 25;2(5):1980–92. doi: 10.1039/d0na00214c (PMC9417705; doi:10.1039/d0na00214c)
Supplement: NA-002-D0NA00214C-s001 [file NA-002-D0NA00214C-s001.pdf]

Electronic Supplementary Material (ESI) for Nanoscale Advances.
This journal is © The Royal Society of Chemistry 2020

Tuning and tracking the growth of gold nanoparticles synthesized using binary surfactant mixtures.

Karthik Raghunathan [†, ‡], *Jibin Antony* [φ, ‡], *Sarmad Munir* [¥], *Jens-Petter Andreassen* [φ], *Sulalit Bandyopadhyay* [φ,*]

† Ugelstad Laboratory, Department of Chemical Engineering, Norwegian University of Science and Technology, N-7491, Trondheim, Norway

φ Department of Chemical Engineering, Norwegian University of Science and Technology, N-7491, Trondheim, Norway

¥ Yara International ASA, Herøya Research Park, Hydrovegen 67, 3936, Porsgrunn, Norway

‡ Authors contributed equally.

Corresponding Author: sulalit.bandyopadhyay@ntnu.no

Table S1. List of experiments performed along with corresponding first-order rate constants.

	**Parameter studied**	**pH of the solution**	**AsA (µl)**	**pH of the solution after AsA**	**k (s^{-1})**	**S(T)EM images**
**Without $AgNO_3$** **Seed = 96µl**	**Effect of pH**	1.5	135	1.5	1.70×10^{-4}	a1
		3.6	135	3	2.93×10^{-3}	a2
		5	135	3.4	3.23×10^{-3}	a3
		7	135	3.4	3.31×10^{-3}	a4
		10	135	5.3	NA	a5
	**Effect of AsA**	NA	68	3.2	NA	NA
		3.6	135	3	2.93×10^{-3}	b1
		NA	200	3.1	3.84×10^{-3}	b2
		NA	270	3.1	3.37×10^{-3}	b3
		NA	400	3	5.01×10^{-3}	b4
**With $AgNO_3$** **$AgNO_3$ = 750 µl** **Seed = 96µl**	**Effect of pH**	1.5	135	1.5	1.05×10^{-4}	c1
		3.6	135	3	1.33×10^{-3}	c2
		5	135	3.3	1.47×10^{-3}	c3
		7	135	3.4	1.58×10^{-3}	c4
		10	135	7.2	2.73×10^{-3}	c5
	**Effect of AsA**	NA	68	3.1	NA	NA
		NA	100	NA	8.08×10^{-4}	NA
		NA	135	3	1.33×10^{-3}	d1

		NA	200	3	1.40×10^{-3}	d2
		NA	270	3	1.47×10^{-3}	d3
		NA	400	2.9	1.94×10^{-3}	d4
**With OA $AgNO_3$ = 750µl OA = 20µl Seed = 96µl**	**Effect of pH**	1.5	135	1.5	1.41×10^{-4}	e1
		3.4	135	3.3	1.92×10^{-3}	e2
		5	135	4.4	2.98×10^{-3}	e3
		7	135	5.5	3.75×10^{-3}	e4
		9.3	135	5.4	3.90×10^{-3}	NA
		10	135	7.1	6.81×10^{-3}	e5
	**Effect of AsA**	NA	68	3.3	1.48×10^{-3}	f1
		NA	135	3.3	1.92×10^{-3}	f2
		NA	200	3.3	2.08×10^{-3}	f3
		NA	270	3.2	2.37×10^{-3}	f4
		NA	400	3.2	2.69×10^{-3}	f5
**Double Seeded $AgNO_3$= 750 µl OA= 30 µl Seed = 200µl**	**Effect of pH**	1.5	135	1.5	7.54×10^{-5}	g1
		3.4	135	3.2	6.08×10^{-4}	g2
		5	135	4.3	7.31×10^{-4}	g3
		7	135	5.3	8.35×10^{-4}	g4
		10	135	6.1	1.80×10^{-3}	g5
	**Effect of AsA**	NA	68	3.3	4.86×10^{-4}	h1
		NA	135	3.2	6.08×10^{-4}	h2
		NA	200	3.2	6.16×10^{-4}	h3

		NA	270	3.2	6.32×10^{-4}	h4
		NA	400	3.1	6.77×10^{-4}	h5

Table S2. List of experiments performed with the data from respective Au NPs

		**AsA (µl)**	**pH of the solution after AsA**	**k (s^{-1})**	**No. of Peaks**	**LSPR (nm)**	**Long Axis (nm)**	**AR**
**Without $AgNO_3$ Seed = 96µl**	**Effect of pH**	135	1.5	1.70×10^{-4}	1	534	64	2.9
		135	3	2.93×10^{-3}	1.5	534	39	1.7
		135	3.4	3.23×10^{-3}	1	531	53	2.7
		135	3.4	3.31×10^{-3}	1.5	532	142	1.3
		135	5.3	NA	1.5	524	36	1.8
	**Effect of AsA**	68	3.2	NA	0	0	0	0
		135	3	2.93×10^{-3}	1.5	534	39	1.7
		200	3.1	3.84×10^{-3}	1	527	46	2.1
		270	3.1	3.37×10^{-3}	1	523	48	2.1
		400	3	5.01×10^{-3}	1	522	34	1.7
**With $AgNO_3$**	**Effect of pH**	135	1.5	1.05×10^{-4}	2	677	42	3.2
		135	3	1.33×10^{-3}	2	720	40	3.2
		135	3.3	1.47×10^{-3}	2	698	39	3.4
		135	3.4	1.58×10^{-3}	2	668	38	2.9

$AgNO_3$ = 750 µl<br>Seed = 96µl		135	7.2	2.73×10^{-3}	3	721	36	2.8
	Effect of AsA	68	3.1	NA	0	0	0	0
		100	NA	8.08×10^{-3}	2	NA	NA	NA
		135	3	1.33×10^{-3}	2	720	40	3.2
		200	3	1.40×10^{-3}	3	756	43	3.4
		270	3	1.47×10^{-3}	3	759	43	3.3
		400	2.9	1.94×10^{-3}	2.5	724	39	3
With OA<br>$AgNO_3$ = 750µl<br>OA = 20µl<br>Seed = 96µl	Effect of pH	135	1.5	1.41×10^{-4}	2	761	51	3.9
		135	3.3	1.92×10^{-3}	2.5	734	44	2.9
		135	4.4	2.98×10^{-3}	2.5	672	34	2.2
		135	5.5	3.75×10^{-3}	2.5	652	27	2.1
		135	5.4	3.90×10^{-3}	1.5	NA	NA	NA
		135	7.1	6.81×10^{-3}	1.5	520	18	1.1
	Effect of AsA	68	3.3	1.48×10^{-3}	3	739	45	2.9
		135	3.3	1.92×10^{-3}	2.5	734	44	2.9
		200	3.3	2.08×10^{-3}	2.5	726	41	2.9
		270	3.2	2.37×10^{-3}	2.5	713	38	2.6
		400	3.2	2.69×10^{-3}	2.5	698	37	2.5
	Effect of pH	135	1.5	7.54×10^{-5}	2	689	121	1.9
		135	3.2	6.08×10^{-4}	3	744	112	1.6
		135	4.3	7.31×10^{-4}	1.5	688	81	1.6

**Double Seeded $AgNO_3$= 750 µl** **OA= 30 µl** **Seed = 200µl**		135	5.3	8.35×10^{-4}	1.5	680	91	1.5
		135	6.1	1.80×10^{-3}	1	536	60	1.1
	**Effect of AsA**	68	3.3	4.86×10^{-4}	2	703	121	1.6
		135	3.2	6.08×10^{-4}	3	744	112	1.6
		200	3.2	6.16×10^{-4}	1.5	735	120	1.6
		270	3.2	6.32×10^{-4}	2	732	114	1.6
		400	3.1	6.77×10^{-4}	1.5	720	111	1.6

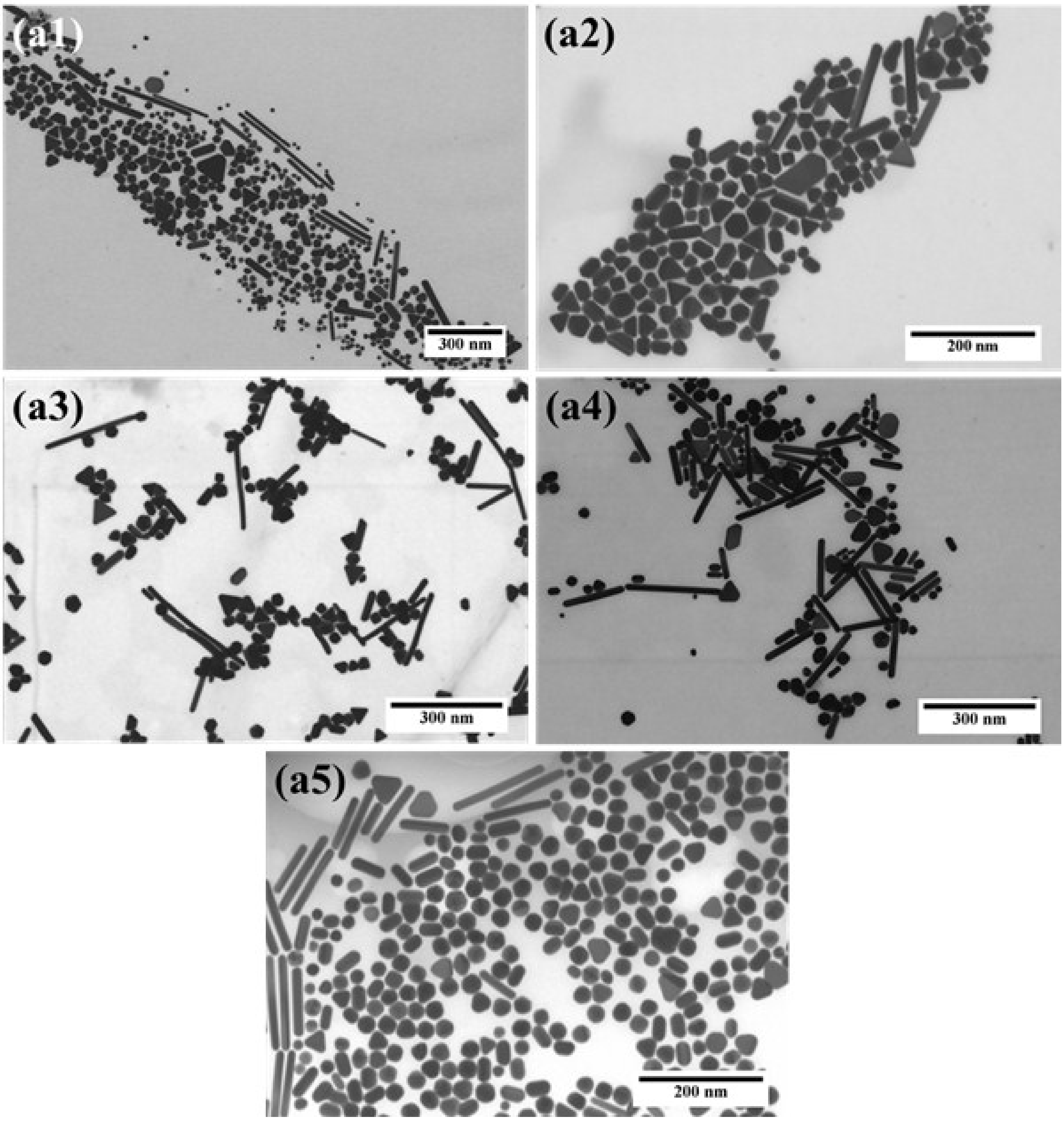

Figure S1. Representative S(T)EM images showing the effect of pH on Au NPs without $AgNO_3$.

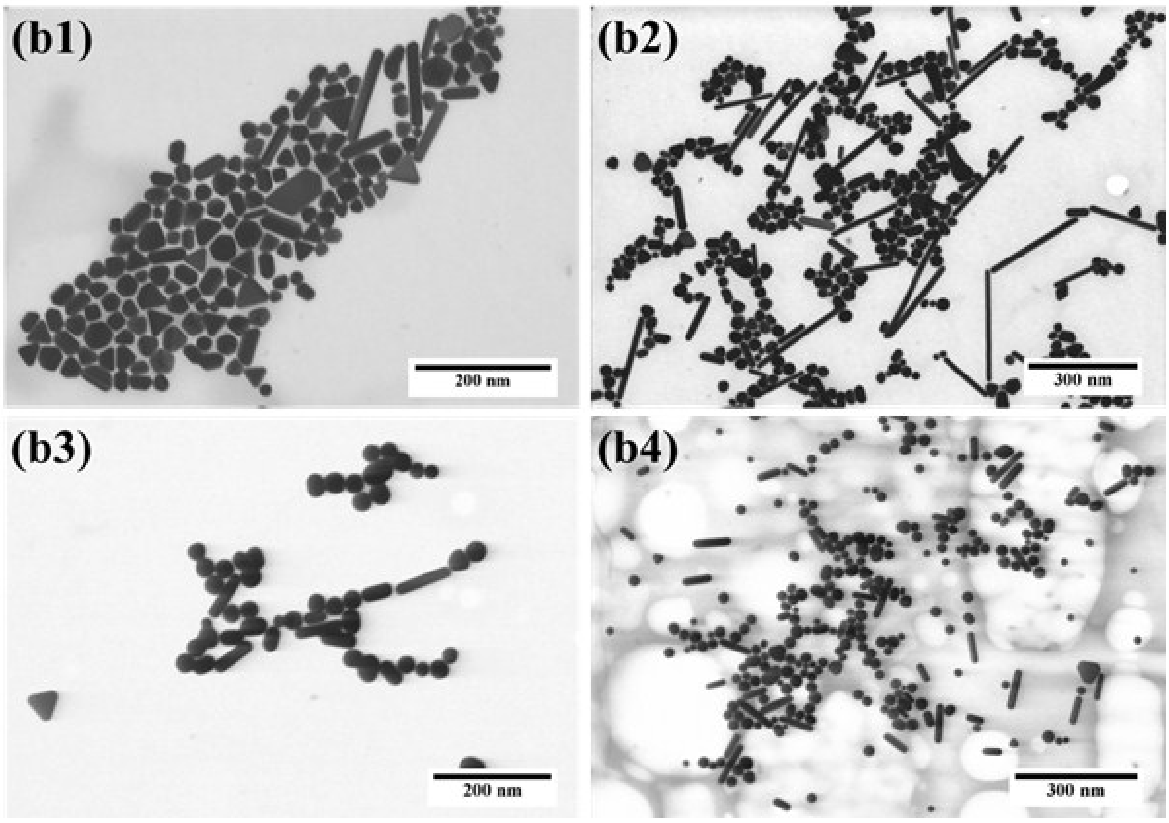

Figure S2. Representative S(T)EM images showing the effect of AsA on Au NPs without $AgNO_3$.

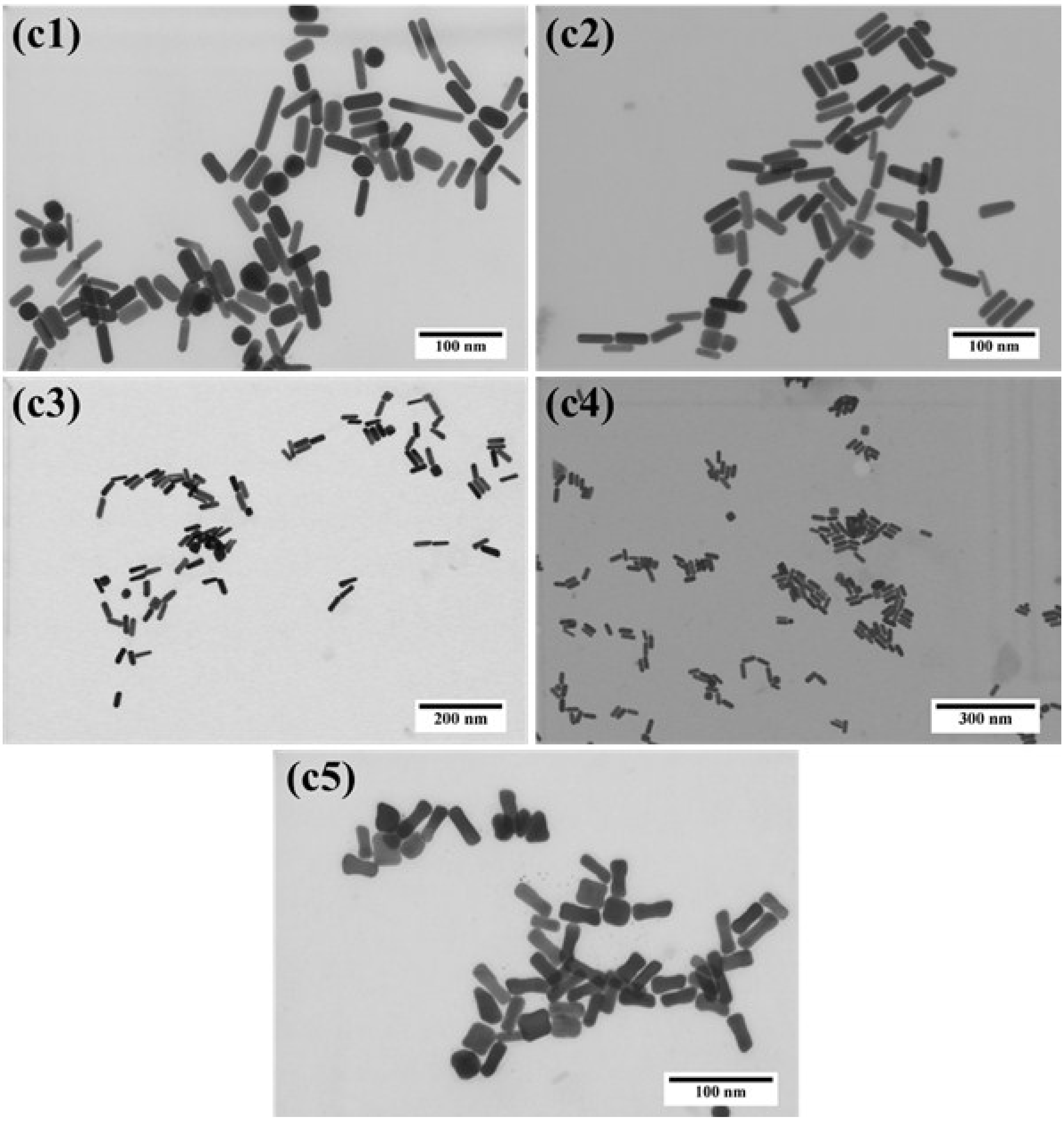

Figure S3. Representative S(T)EM images showing the effect of pH on Au NPs with $AgNO_3$.

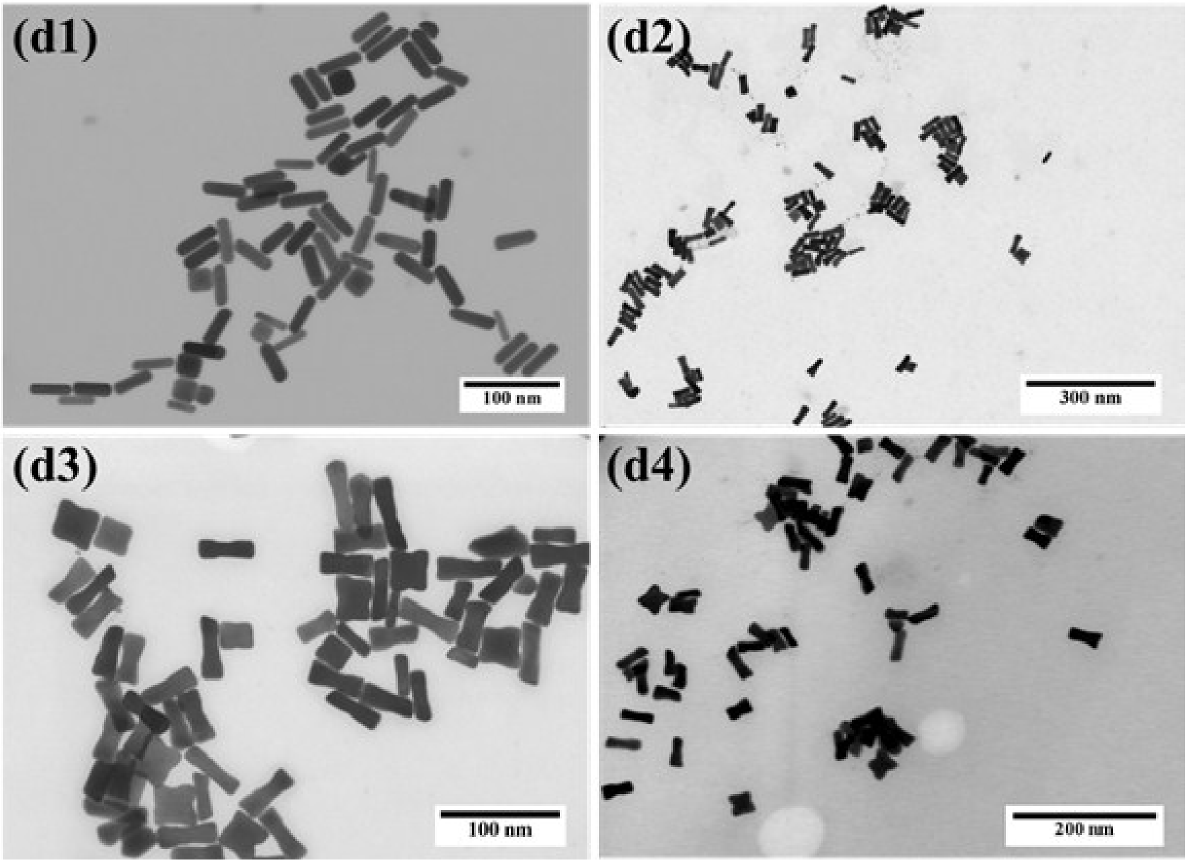

Figure S4. Representative S(T)EM images showing the effect of AsA on Au NPs with $AgNO_3$.

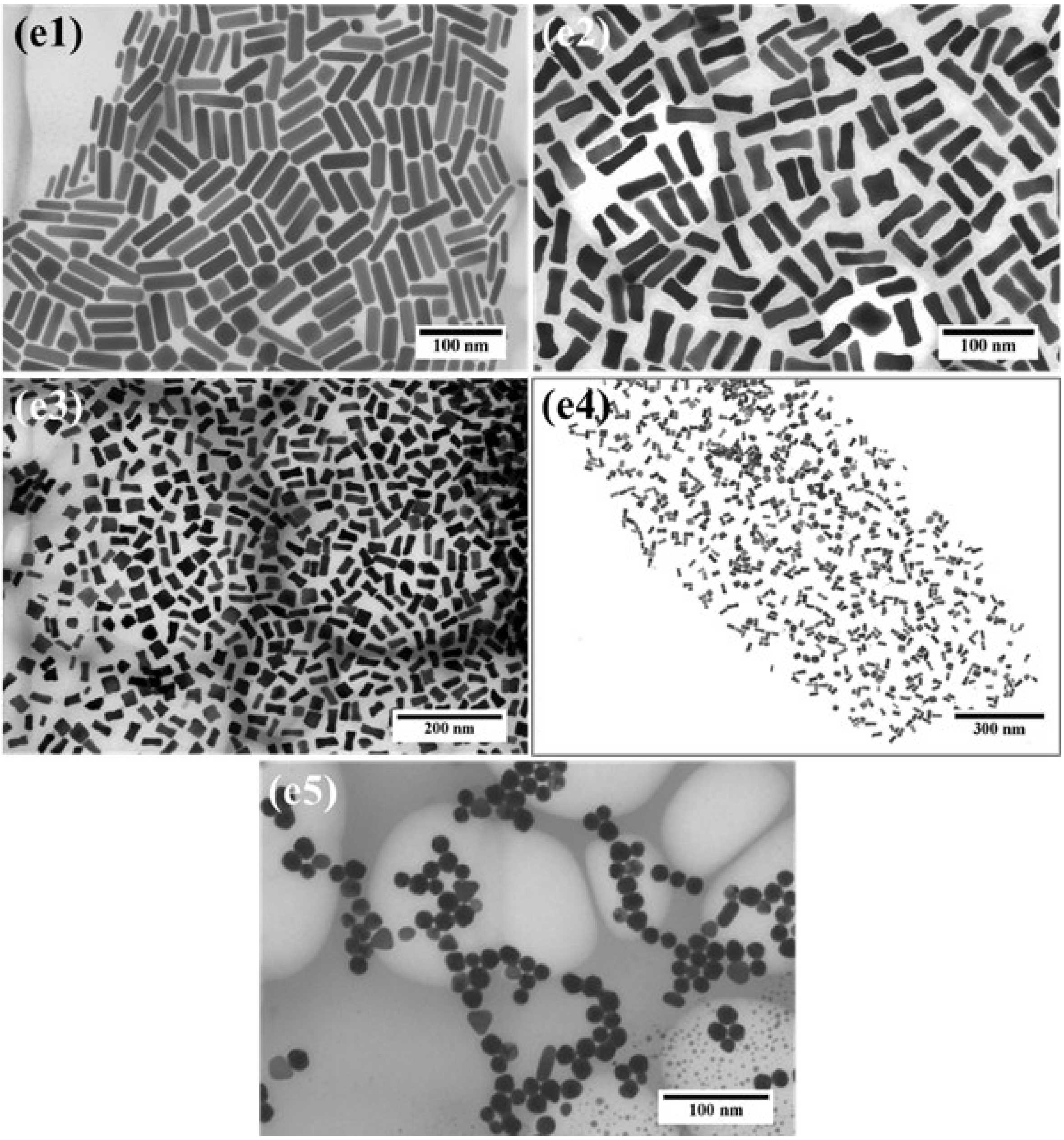

Figure S5. Representative S(T)EM images showing the effect of pH on Au NPs with OA.

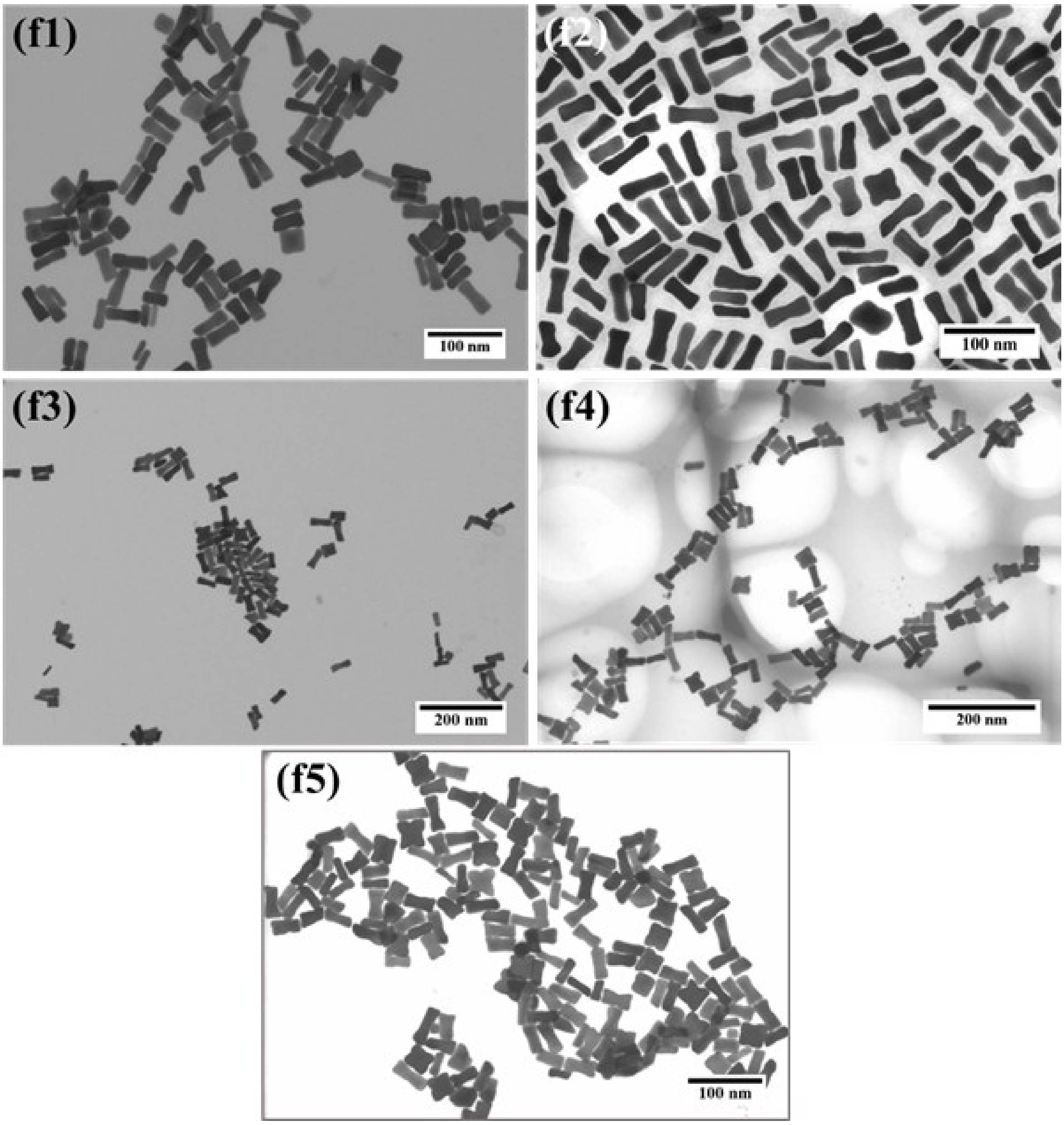

Figure S6. Representative S(T)EM images showing the effect of AsA on Au NPs with OA.

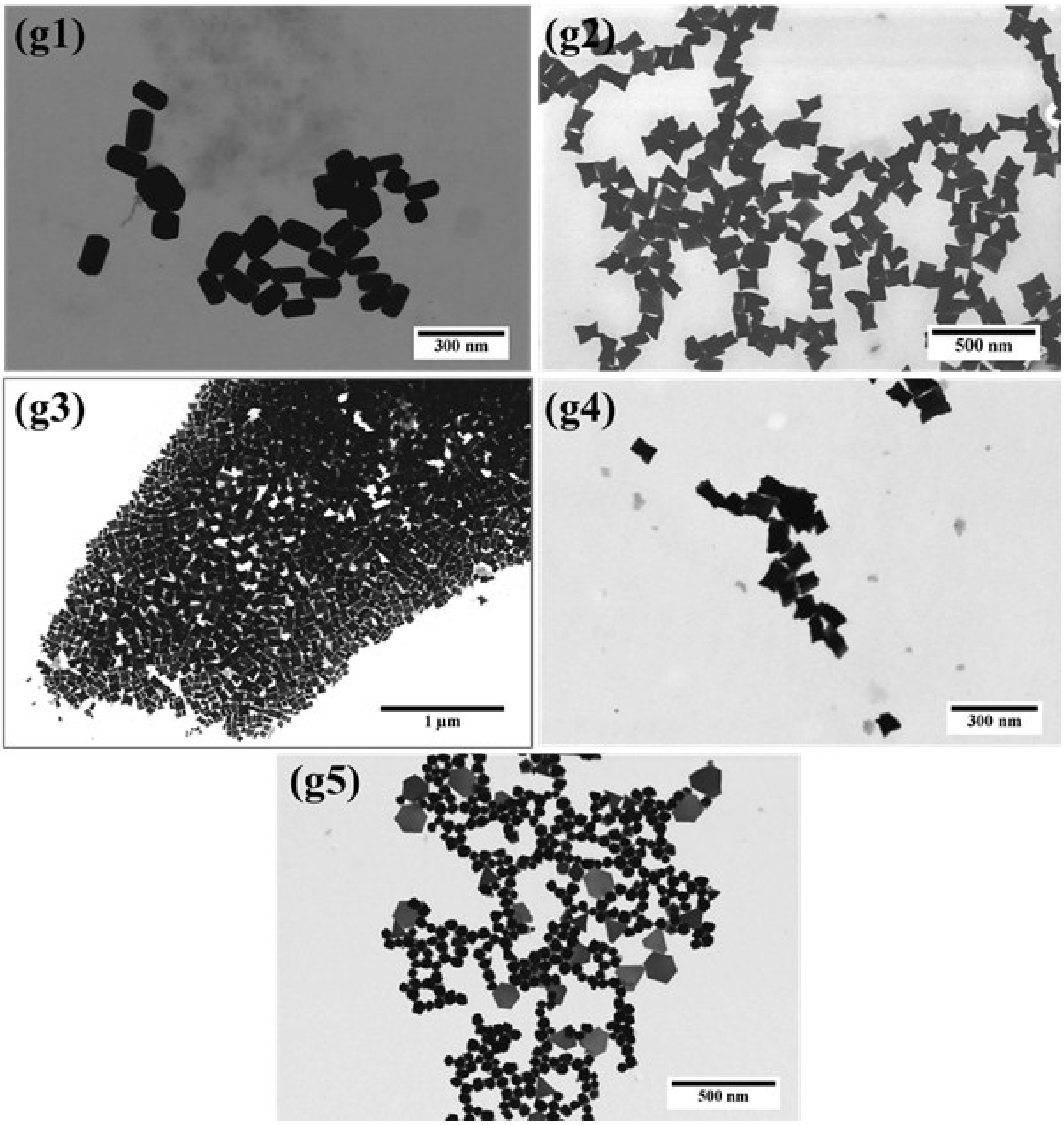

Figure S7. Representative S(T)EM images showing the effect of pH on double seeded Au NPs.

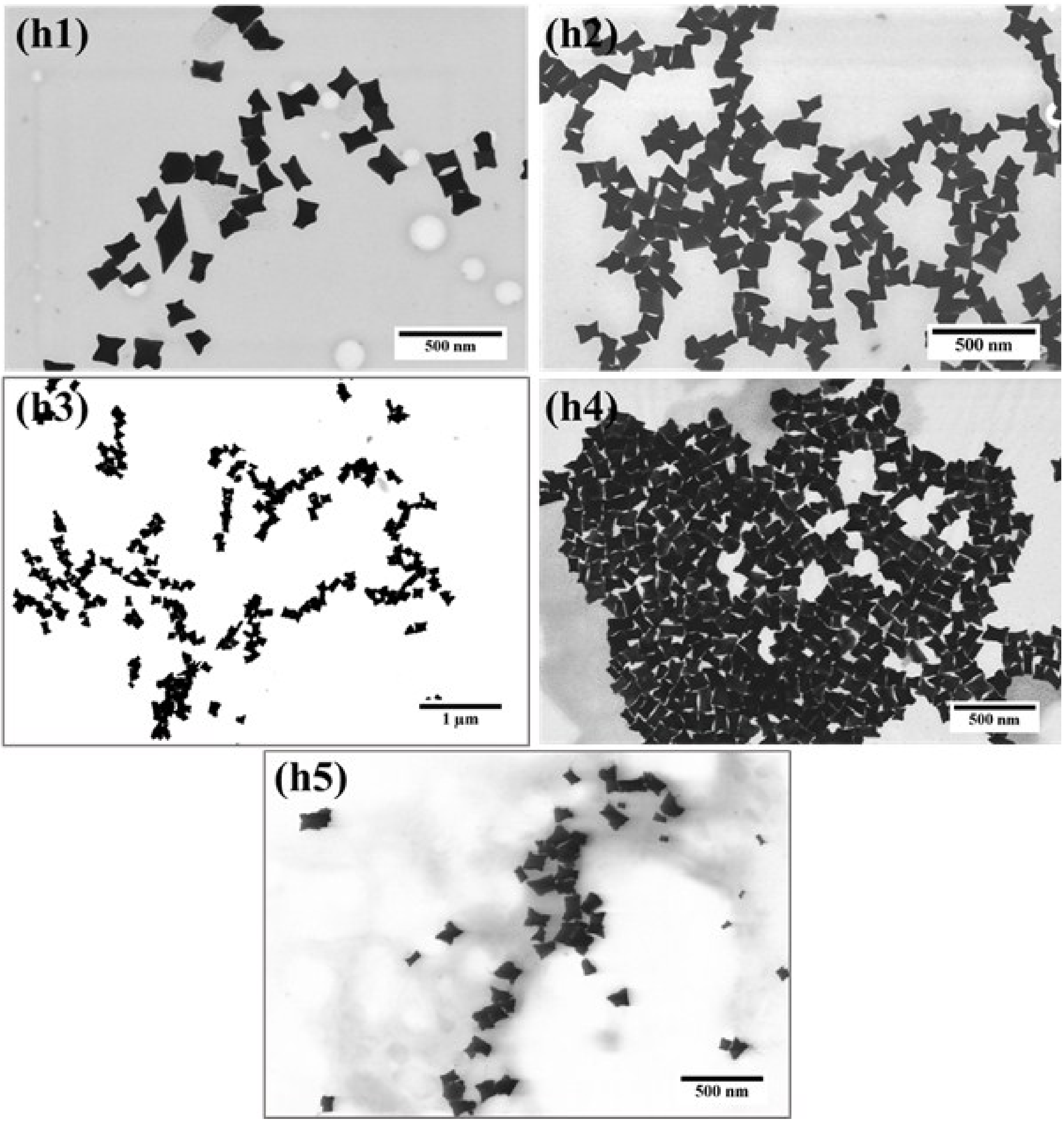

Figure S8. Representative S(T)EM images showing the effect of AsA on double seeded Au NPs.

Table S3. Fit parameters obtained for different conditions

**Parameters**	**One hour**	**Completion of Reaction**
**a**	1.03	1.24
**b**	259	259
**x0**	1051	1051
**y0**	-3.06×10^{-2}	-3.70×10^{-2}

Calculating kinetics of growing NPs

A robust, repeatable method to calculate kinetics of growing NPs wAS used in the experiments mentioned in Table S1. The method development is laid out in this section. Figure 5(a) shows some of the UV-Vis measurements of growing NRs taken at different time points from 0 to 60 mins from the start of the reaction (addition of seeds). Figure 5(b) shows the area under all the curves obtained from UV-Vis. The area plot shows that there is an initial lag phase where the area increases slightly. This is followed by exponential growth and finally reaches a region where the area remains constant. The time taken to reach this final stage varies for different NPs. The area under the curve obtained was normalized against the area obtained after completion of the reaction. The fit parameters obtained were compared against the fit parameters from the area obtained once the area starts to remain constant. The UV-Vis measurements were done until a constant area is obtained or at least for one hour. The fit parameters obtained when the normalization is done using the area after one hour and after the completion of reaction are shown in Table S3.

For ease of analysis, normalization with area obtained after reaching constant values can be performed. Only a small volume is used in UV-Vis measurements (3.5 ml from ~30ml), normalization was done using areas obtained from the cuvette measurements and from bulk (3.5ml was withdrawn after one hour from the reaction mixture and measured in UV-Vis). The

parameters obtained from curve fits were the same indicating that the cuvette is a representation of reactions happening in the bulk.

Table S4. Comparison between fit parameters obtained for different wavelength ranges for NRs

**Parameters**	**Transverse axis (400-650nm)**	**Longitudinal Axis (650-900nm)**	**Au NPs (400-900nm)**
**a**	9.79×10^{-1}	9.99×10^{-1}	9.86×10^{-1}
**b**	138	121	128
**x_0**	593	632	615
**y_0**	2.01×10^{-2}	1.40×10^{-2}	1.39×10^{-2}

Table S5. First-order rate constants k comparing shelf life of chemicals

	**k (s^{-1}) – one-day shelf life**	**k (s^{-1})**
**Without AgNO3**	$2.58\times10^{-3} \pm 1.47\times10^{-5}$	$3.42\times10^{-3} \pm 1.05\times10^{-3}$
**With AgNO3**	$1.29\times10^{-3} \pm 7.92\times10^{-5}$	$1.09\times10^{-3} \pm 2.28\times10^{-4}$
**With OA**	$2.27\times10^{-3} \pm 1.64\times10^{-4}$	$2.34\times10^{-3} \pm 9.61\times10^{-4}$

Table S6. Comparison between sigmoidal fit and exponential fit for various reaction conditions

Experiment	Sigmoidal fit	Exponential fit
**Without AgNO3**	2.59×10^{-3}	2.52×10^{-3}
**With AgNO3**	1.30×10^{-3}	1.28×10^{-3}
**With OA**	2.28×10^{-3}	2.26×10^{-3}
**Double Seeded**	5.61×10^{-4}	5.51×10^{-4}

Comparison between sigmoidal fit and exponential fit

The sigmoidal nature of curves obtained for these NPs shows an initial exponential growth. The time taken for the area under the curves to reach the maximum values is short when compared to the total reaction time. For a quick calculation of kinetics or for the NPs whose time progression is known, the UV-Vis measurements can be done for the initial growth and exponential rise to maximum fit can be used. The equation for this fit:

$$y = y_0 + a\exp(bx)$$

The rate constants calculated by fitting sigmoidal fit and the exponential fit for different experiments are shown in Table S5. Although similar results are obtained, the sigmoidal fit is recommended when calculating the kinetics for the first time for a given experimental condition as this method includes more data points.

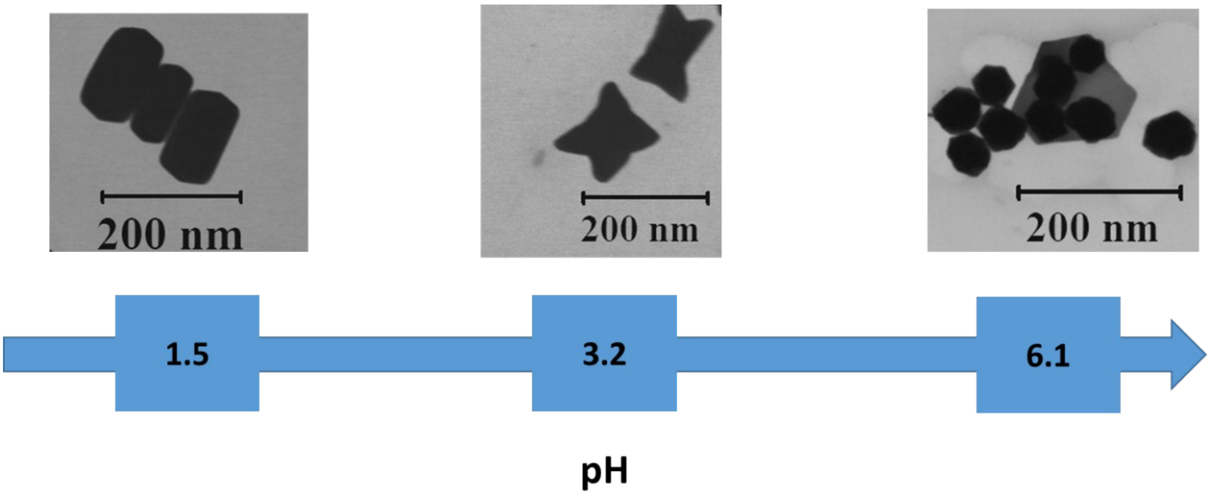

Figure S9. Representative S(T)EM images showing the effect of pH on double seeded Au NPs.

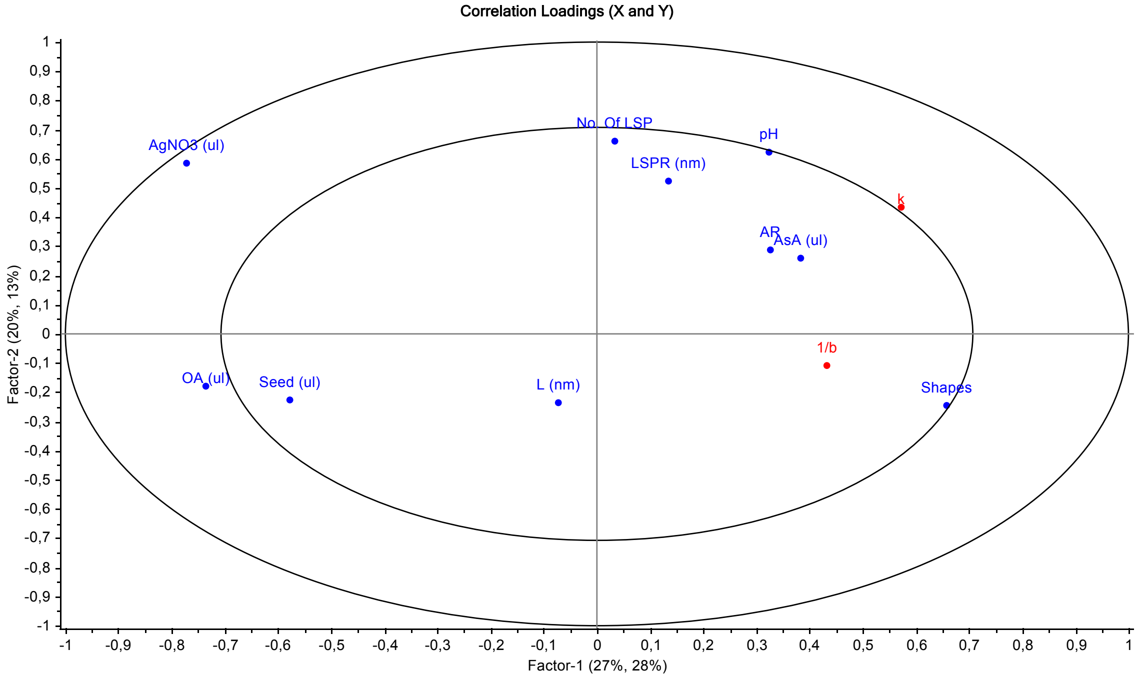

Figure S10. Correlation loadings diagram with k and 1/b as output

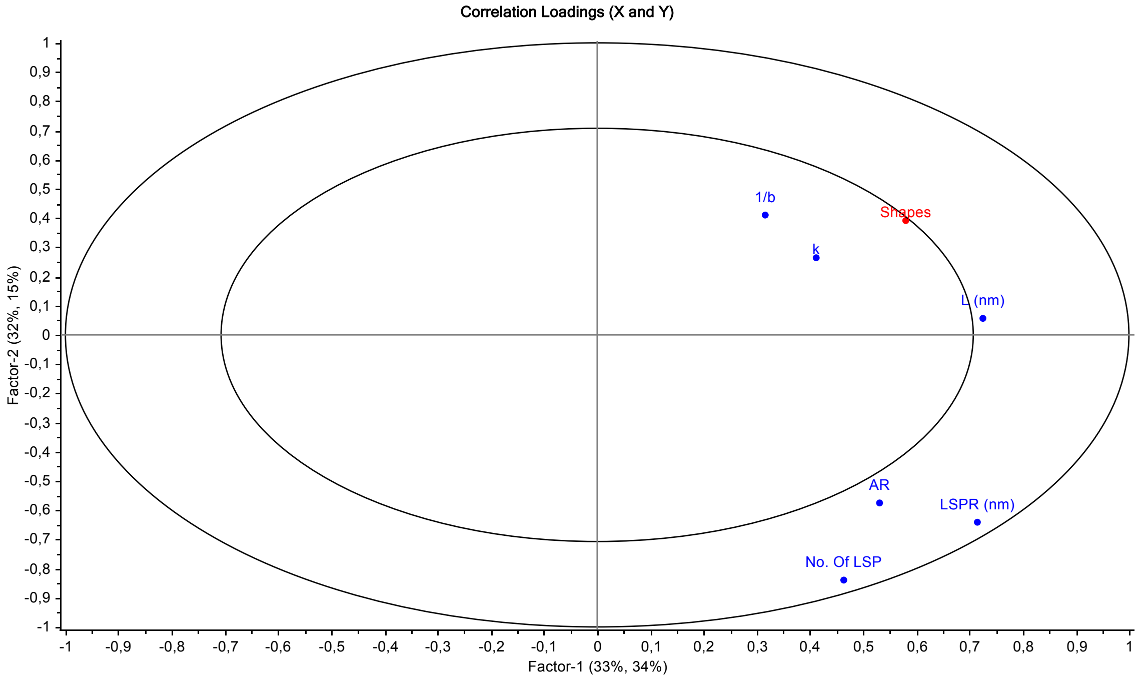

Figure S11. Correlation loadings diagram with shape as output

The correlation loading plots S10 and S11 are based on Table S2. For each model used by UnScramblerX for a given dataset, the variable in the outer ellipse explain 100% of the variations in the data whereas the variables in the inner ellipse explain 50% of the variance. Although from Figure S11 1/b and k appear to be equidistant from shape, in Figure S10 k can explain the variance in the dataset better than 1/b for the model. The results corroborate with the low standard deviations observed in k for repeats when compared with 1/b obtained for the same experiments, shown in Table S7.

	**k (s^{-1})**	**1/b (s^{-1})**
**Without AgNO3**	$2.58\times10^{-3}\pm1.47\times10^{-5}$	$31.97\times10^{-2} \pm 7.26\times10^{-4}$
**With AgNO3**	$1.29\times10^{-3}\pm7.92\times10^{-5}$	$9.11\times10^{-3} \pm 3.04\times10^{-4}$
**With OA**	$2.27\times10^{-3}\pm1.64\times10^{-4}$	$1.57\times10^{-2} \pm 1.57\times10^{-3}$

Table S7. Comparison between k and (1/b) for different synthesis conditions

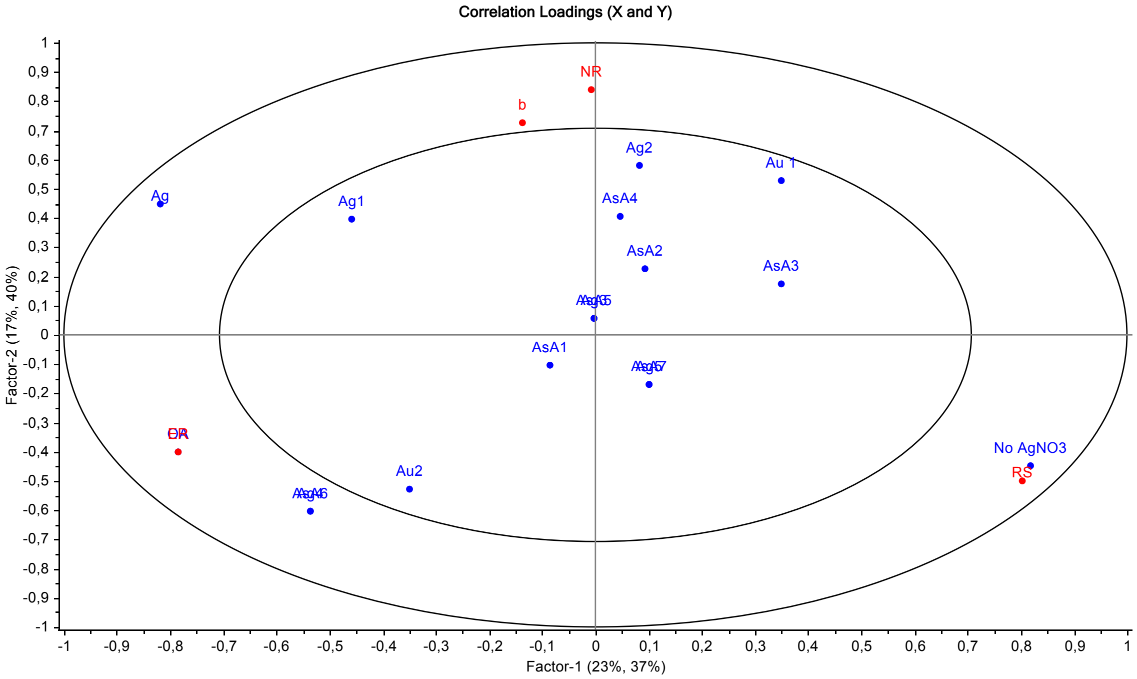

Correlation Loadings (X and Y)
NR
b
Ag2
Au 1
Ag
Ag1
AsA4
AsA2
AsA3
AsA1
Au2
No AgNO3
RS
Factor-2 (17%, 40%)
Factor-1 (23%, 37%)

a

Parameter Estimates

Term	Estimate	Std Error	t Ratio	Prob>|t|
Intercept	0.0081264	0.001627	5.00	<.0001*
LSPR	-1.648e-5	2.919e-6	-5.65	<.0001*
No of LSPR peaks	0.0011226	0.00037	3.03	0.0051*
AsA	4.7286e-6	1.648e-6	2.87	0.0076*
pH	0.000487	0.000115	4.23	0.0002*

b

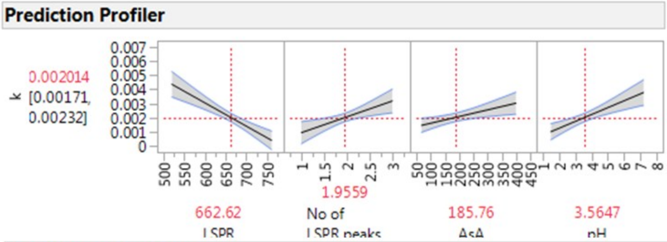

c

Parameter Estimates

Term	Estimate	Std Error	t Ratio	Prob>|t|
Intercept	56.769004	41.87957	1.36	0.1861
AgNO3	-0.061936	0.017196	-3.60	0.0012*
k	-13127.9	2445.734	-5.37	<.0001*
LSPR	0.2333769	0.076483	3.05	0.0049*
AR	-37.83461	4.496898	-8.41	<.0001*

d

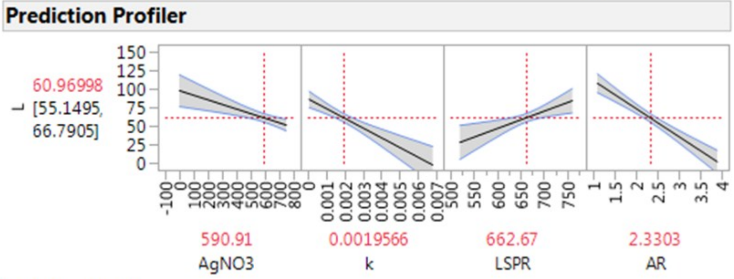

e

Parameter Estimates

Term	Estimate	Std Error	t Ratio	Prob>|t|
Intercept	282.50471	25.45569	11.10	<.0001*
OA	1.2682484	0.61445	2.06	0.0487*
AgNO3	0.106965	0.02522	4.24	0.0002*
AsA	0.1298227	0.045139	2.88	0.0078*
No of LSPR peaks	45.860822	9.428898	4.86	<.0001*
L	0.8998596	0.15581	5.78	<.0001*
AR	56.133334	8.629902	6.50	<.0001*

f

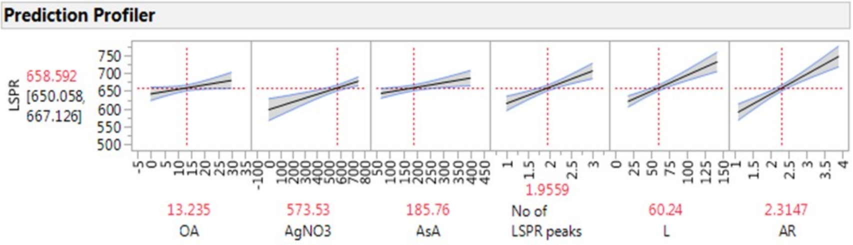

g

Parameter Estimates

Term	Estimate	Std Error	t Ratio	Prob>|t|
Intercept	-3.518514	0.707808	-4.97	<.0001*
k	162.42763	52.9271	3.07	0.0044*
LSPR	0.0077643	0.000959	8.10	<.0001*

h

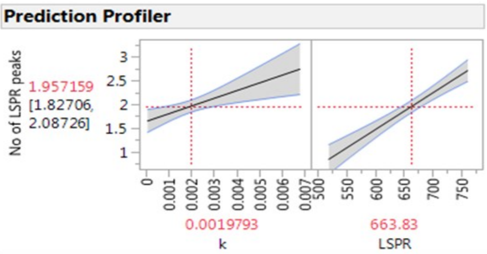

i

Parameter Estimates

Term	Estimate	Std Error	t Ratio	Prob>|t|
Intercept	0.82448	0.496434	1.66	0.1079
OA	-0.02441	0.004948	-4.93	<.0001*
AsA	-0.001541	0.000521	-2.96	0.0062*
pH	-0.209796	0.040325	-5.20	<.0001*
LSPR	0.0052995	0.000618	8.58	<.0001*
L	-0.010681	0.001784	-5.99	<.0001*

j

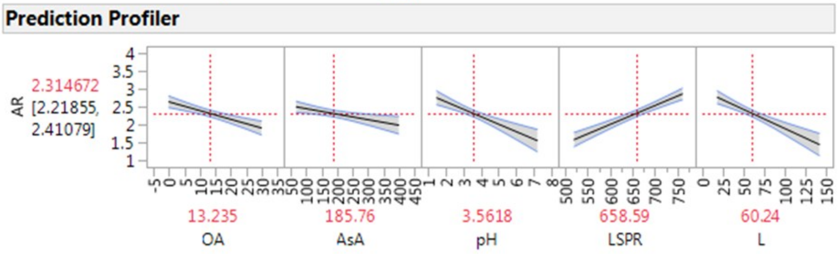

Figure S12. Correlation loadings diagram for different shapes and fit parameter b

Figure S13. Multivariate Linear Regression obtained from JMP (a) and (b) Parameter estimated and predictor profile when y variable is k. (c) and (d) Parameter estimated and predictor profile when y variable is L. (e) and (f) Parameter estimated and predictor profile when y variable is LSPR. (g) and (h) Parameter estimated and predictor profile when y variable is No of LSPR peaks. (i) and (j) Parameter estimated and predictor profile when y variable is AR.

Linear regression analysis was performed using JMP. The following are the predictor estimates and predictor profiler for the different y variables obtained from JMP software. The predictor estimate shows the modal with its standard deviation along with its confidence interval. The predictor profiler shows the behavior of the model.
